# An Analysis of Thaumarchaeota Populations from the Northern Gulf of Mexico

**DOI:** 10.3389/fmicb.2013.00072

**Published:** 2013-04-09

**Authors:** Bradley B. Tolar, Gary M. King, James T. Hollibaugh

**Affiliations:** ^1^Department of Marine Sciences, University of GeorgiaAthens, GA, USA; ^2^Department of Microbiology, University of GeorgiaAthens, GA, USA; ^3^Department of Biological Sciences, Louisiana State UniversityBaton Rouge, LA, USA

**Keywords:** thaumarchaeota, euryarchaeota, nitrite-oxidizing Bacteria, hypoxia, Gulf of Mexico, ammonia monooxygenase, acetyl-CoA/propionyl-CoA carboxylase, 4-hydroxybutyryl-CoA dehydratase

## Abstract

We sampled Thaumarchaeota populations in the northern Gulf of Mexico, including shelf waters under the Mississippi River outflow plume that are subject to recurrent hypoxia. Data from this study allowed us to: (1) test the hypothesis that Thaumarchaeota would be abundant in this region; (2) assess phylogenetic composition of these populations for comparison with other regions; (3) compare the efficacy of quantitative PCR (qPCR) based on primers for 16S rRNA genes (*rrs*) with primers for genes in the ammonia oxidation (*amoA*) and carbon fixation (*accA*, *hcd*) pathways; (4) compare distributions obtained by qPCR with the relative abundance of Thaumarchaeota *rrs* in pyrosequenced libraries; (5) compare Thaumarchaeota distributions with environmental variables to help us elucidate the factors responsible for the distributions; (6) compare the distribution of Thaumarchaeota with Nitrite-Oxidizing Bacteria (NOB) to gain insight into the coupling between ammonia and nitrite oxidation. We found up to 10^8^ copies L^−1^ of Thaumarchaeota *rrs* in our samples (up to 40% of prokaryotes) by qPCR, with maximum abundance in slope waters at 200–800 m. Thaumarchaeota *rrs* were also abundant in pyrosequenced libraries and their relative abundance correlated well with values determined by qPCR (*r*^2^ = 0.82). Thaumarchaeota populations were strongly stratified by depth. Canonical correspondence analysis using a suite of environmental variables explained 92% of the variance in qPCR-estimated gene abundances. Thaumarchaeota *rrs* abundance was correlated with salinity and depth, while *accA* abundance correlated with fluorescence and pH. Correlations of Archaeal *amoA* abundance with environmental variables were primer-dependent, suggesting differential responses of sub-populations to environmental variables. Bacterial *amoA* was at the limit of qPCR detection in most samples. NOB and Euryarchaeota *rrs* were found in the pyrosequenced libraries; NOB distribution was correlated with that of Thaumarchaeota (*r*^2^ = 0.49).

## Introduction

The Mississippi River outflow forms a surface plume up to 10 m thick upon entering the northern Gulf of Mexico. Stratification and nutrient (especially nitrogen) enrichment of river water (Turner et al., 2006) lead to elevated primary production in the plume and thus to increased organic matter deposition 10 to 100 km away from river discharge sites (Rabalais et al., 2002; Green et al., 2008). Decomposition of this organic matter is thought to contribute to the formation of a recurrent hypoxic zone in the northern Gulf of Mexico that profoundly affects the ecology, fisheries biology, and geochemistry of the region (Rabalais et al., 2002; Dagg et al., 2007; Cai et al., 2011). Intermittent hypoxia ([O_2_] ≤ 2 mL/L or ∼90 μM; Diaz and Rosenberg, 2008) begins to develop in February and typically is most pronounced from mid-May to mid-September (Rabalais et al., 2010).

Processes such as coupled nitrification/denitrification that remove excess fixed nitrogen affect primary production and thus may be important determinants of the extent and duration of hypoxia. Ammonia oxidation is the first step in the biogeochemical pathway leading to denitrification. Members of the β- and γ-subdivisions of the Proteobacteria (Ammonia-Oxidizing Bacteria, AOB) and Marine Group 1 Archaea (Ammonia-Oxidizing Archaea, AOA) can grow chemoautotrophically by oxidizing ammonia to nitrite (Ward, 2011). The nitrite produced can be oxidized further to nitrate by Nitrite-Oxidizing Bacteria (NOB) and then denitrified (Jetten, 2001; Francis et al., 2007; Ward et al., 2009).

Ammonia monooxygenase genes (*amoA*) from AOA have been observed in marine environments at 10–1,000 times greater abundance than the *amoA* homolog from AOB, suggesting that the AOA play a key role in the marine nitrogen cycle (Francis et al., 2005, 2007; Wuchter et al., 2006; Mincer et al., 2007; Prosser and Nicol, 2008; Santoro et al., 2010; Ward, 2011). Currently, the functional guild of marine AOA includes members of the Marine Group 1 Archaea (DeLong, 1992; Fuhrman et al., 1992) and organisms related to a deeply branching clade (pSL12) of hot-spring crenarchaeotes (Barns et al., 1996) that are predicted to possess the *amoA* gene (Mincer et al., 2007). Genomic evidence suggests that Marine Group 1 Archaea and related organisms from benthic, terrestrial, and hot-spring habitats, as well as a sponge symbiont, should be assigned to a new phylum, the Thaumarchaeota, within the kingdom Archaea (Brochier-Armanet et al., 2008; Spang et al., 2010; Kelly et al., 2011). We use this term hereinafter in place of “Marine Group 1 Archaea.”

Pelagic marine Thaumarchaeota are typically most abundant below ∼100 m depth in the water column (DeLong, 1992; Fuhrman et al., 1992; Massana et al., 1997; Karner et al., 2001; Mincer et al., 2007; Church et al., 2010; Santoro et al., 2010), in surface waters at higher latitudes and polar oceans (Massana et al., 1998; Murray et al., 1998, 1999b; Church et al., 2003; Alonso-Sáez et al., 2008; Kalanetra et al., 2009), and in hypoxic regions and oxygen minimum zones (OMZs; [O_2_] ≤ 0.5 mL/L or ≤22 μM; Levin, 2003) such as the Black Sea, Baltic Sea, Gulf of California, Arabian Sea, and the eastern tropical Pacific Ocean (Coolen et al., 2007; Lam et al., 2007, 2009; Beman et al., 2008; Labrenz et al., 2010; Molina et al., 2010). Previous studies are contradictory but have pointed to environmental factors such as salinity, light, temperature, ammonium, oxygen, and sulfide as major determinants of this distribution (e.g., Murray et al., 1999a; Caffrey et al., 2007; Santoro et al., 2008; Bernhard et al., 2010; Gubry-Rangin et al., 2010; reviewed in Prosser and Nicol, 2008; Erguder et al., 2009; Nicol et al., 2011; Ward, 2011). Bacterial or phytoplankton biomass has also been thought to influence Thaumarchaeota distributions (Murray et al., 1999a,b; Church et al., 2003), perhaps through competition for resources.

One of the goals of the present study was to quantify the distribution of AOA in the northern Gulf of Mexico in the area influenced by the Mississippi River plume and recurrent hypoxia. We hypothesized that ammonia oxidizers would be abundant there because of the high riverine nitrogen loading to the region and the importance of respiration (Cai et al., 2011), and thus presumably nitrogen regeneration, in the region experiencing hypoxia. We also hypothesized that AOA would dominate ammonia oxidizer populations at pelagic stations, although AOB were found to be more abundant than AOA in sediments from Weeks Bay, Alabama (Caffrey et al., 2007). To test these hypotheses, we determined AOA and AOB distributions by quantitative PCR (qPCR) measurements of the abundance of *rrs* and *amoA* genes. We also pyrosequenced *rrs* genes from our samples as an independent check on distributions based on qPCR data. A second goal was to analyze variation in sequences of *rrs* and compare this to genes from two metabolic pathways that are important to AOA, ammonia oxidation and carbon fixation, to provide a more highly resolved description of the composition of Thaumarchaeota populations than can be obtained from analyses of single genes. AOA can grow autotrophically (Könneke et al., 2005) using the 3-hydroxypropionate/4-hydroxybutyrate pathway (Berg et al., 2007). The potential for AOA autotrophy can be detected in the environment using primers targeting the genes in this pathway, notably acetyl-CoA/propionyl-CoA carboxylase (*accA*; Yakimov et al., 2009) and 4-hydroxybutyryl-CoA dehydratase (*hcd*; Offre et al., 2011). We tested both of these primer sets with our samples. We compared the phylogenetic diversity present in their amplicons with diversity represented in amplicons from more widely used primer sets for *amoA* and *rrs*. We then used *rrs* sequences from the pyrosequencing effort to extend phylogenetic inferences based on analyses from samples taken at one station more broadly across the study area. A third goal was to investigate the relationship between Thaumarchaeota distributions and environmental variables to provide insight into the factors controlling their distribution. Pyrosequencing data were also used to compare the distribution of NOB with AOA to gain insights into the coupling between these two steps of nitrification.

## Materials and Methods

### Sample collection and DNA extraction

**Figure 1:**
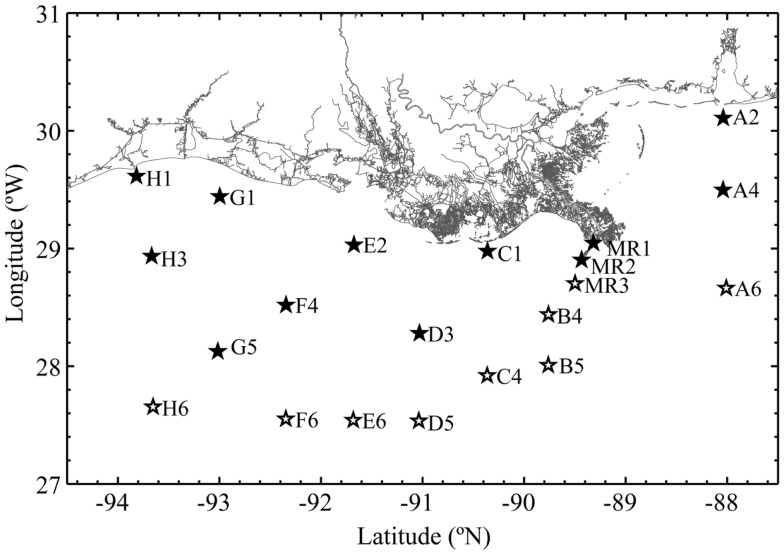
**Stations occupied during the GulfCarbon 5 cruise, March 10–21, 2010**. Inshore stations represented with a filled star; offshore stations have an open star.

Samples were collected during the *R/V Cape Hatteras* GulfCarbon 5 cruise in the northern Gulf of Mexico (30°07′N, 088°02′W to 27°39′N, 093°39′W; Figure 1) from March 10–21, 2010. Samples were collected using Niskin bottles and a General Oceanics rosette sampling system equipped with an SBE25 CTD and sensors for [O_2_], beam attenuation (turbidity), and relative fluorescence (calibrated to chlorophyll a equivalents). The [O_2_] sensor was cross-calibrated against Winkler titrations of [O_2_] in samples collected at fixed depths. pH data were collected using a glass electrode by W.-J. Huang of Dr. W.-J. Cai’s group. Euphotic depth (defined as 1% PAR, 400–700 nm) was calculated for each station from Aqua MODIS satellite data using an average of the Lee and Morel models^1^ by H. Reader and C. Fichot. Nutrient data were collected at some of the station/depths we sampled by Dr. S. Lohrenz’s group. Since nutrient sample collections were biased in favor of near-surface samples on the continental shelf, these data were used only in BEST analysis (see Appendix). Approximately 1 L of water from each Niskin bottle was pressure filtered (at ∼60 kPa) through 0.22 μm Durapore filters (Millipore); filters were frozen in 2 mL of lysis buffer (0.75 M sucrose, 40 mM EDTA, 50 mM Tris; pH 8.3). DNA was extracted by enzymatic hydrolysis with lysozyme (50 mg mL^−1^), proteinase K (20 mg mL^−1^), and sodium dodecyl sulfate (100 μL of a 10% solution), and then purified by phenol-chloroform extraction as described previously (Bano and Hollibaugh, 2000).

### Quantitative PCR

Quantitative PCR was performed using an iCycler iQ™ Real-Time qPCR detection system (Bio-Rad) and the primers listed in Table A1 in Appendix. qPCR reactions were run in triplicate with standards made from environmental amplicons as described in the “Methods” in Appendix. TaqMan^®^ (Applied Biosystems) chemistry was used to detect amplification of Bacteria and Thaumarchaeota 16S rRNA genes (*rrs*) following Kalanetra et al. (2009); all other amplifications were detected using SYBR^®^ Green Supermix (Bio-Rad). We compared two primer sets for detecting Archaeal *amoA*: Arch-amoA-for and Arch-amoA-rev (“Wuchter primers”; Wuchter et al., 2006) and ArchamoAF and ArchamoAR (“Francis primers”; Francis et al., 2005). Reactions using the Wuchter primers were set up as described in Kalanetra et al. (2009), while PCR conditions for the Francis primers followed Santoro et al. (2010), except that SYBR^®^ Green Supermix (Bio-Rad) was used with no additional MgCl_2_. Amplification of pSL12 *rrs* followed Mincer et al. (2007), with the number of amplification cycles reduced to 40 to prevent quenching of the fluorescence signal. Archaeal *accA* genes were amplified following Yakimov et al. (2009) with shorter cycle lengths (Hu et al., 2011). Specificity of SYBR^®^ Green reactions was confirmed by melting curve analysis; *accA* amplicons were also checked by sequencing clones created with qPCR primers Crena_529F and Crena_981R (Yakimov et al., 2009). We also tested published primers for *hcd* genes (Offre et al., 2011), but found that non-specific amplification rendered them unsuitable for qPCR with our samples (see Appendix). Inhibition of qPCR reactions was tested using dilutions of DNA 10–1,000× with the Bacterial *rrs* qPCR assay; samples that showed higher copy number than expected from typical dilution were determined to have PCR inhibitors present and run at the dilution which gave the highest copy number for all other gene assays. Calculations of gene abundance and ratios are discussed in the “Methods” in Appendix, and qPCR efficiencies for reactions are reported in Table A1 in Appendix.

### Phylogenetic analysis

We sequenced cloned *rrs*, *amoA*, and *accA* amplicons to obtain phylogenetic descriptions of the Thaumarchaeota populations in the study area and to verify specificity of qPCR reactions. Libraries were generated from samples collected at Station D5, located on the southern edge of the area influenced by the Mississippi River plume and over the continental slope (Figure 1) using methods described previously (Kalanetra et al., 2009) and summarized below. This station was chosen for its depth and as representative of slope stations influenced by hypoxia. We compared samples from different depths at this station as others (e.g., Lam et al., 2007; Beman et al., 2008; Kalanetra et al., 2009; Church et al., 2010; Santoro et al., 2010) have shown segregation of Thaumarchaeota populations by depth. *rrs* and *amoA* were amplified from DNA collected at 100 and 200 m, while *accA* amplicons were generated from samples collected at 2, 50, 100, 200, and 450 m to test the *accA* primer set across a wider depth range. PCR amplifications of Archaeal *rrs*, *amoA*, and *accA* used the primers listed in Table A1 in Appendix. Three separate amplifications were pooled to minimize potential PCR bias and electrophoresed on a 1% agarose gel. The band of the expected DNA product size was excised, extracted and purified using the QIAquick^®^ Gel Extraction Kit (QIAGEN), and incorporated into a TOPO 4 vector (Invitrogen) prior to cloning using chemically competent TOP10 *E. coli* cells with the TOPO TA cloning kit (Invitrogen) following the manufacturer’s instructions. Clones from each library were selected randomly and sequenced (Genewiz, Inc.) using the plasmid primer M13F(−21). Euryarchaeota *rrs* sequences were identified by BLAST (Zhang et al., 2000) and not analyzed further.

Sequences were inspected manually and checked for vector contamination using Geneious v. 5.41^2^. Thaumarchaeota *rrs* sequences were checked for chimeras using Bellerophon (Huber et al., 2004); three chimeric sequences were identified and discarded. Nucleotide and inferred amino acid sequences for *amoA* and *accA* were aligned in Geneious, while *rrs* nucleotide sequences were first aligned using the Silva aligner (v.1.2.5; Pruesse et al., 2007) and then imported into ARB (v. 5.2; Ludwig et al., 2004), manually trimmed, and inspected for alignment errors. Sequences obtained from these libraries have been deposited in GenBank (NCBI) under accession numbers KC330756 to KC330822 (*rrs* – Thaumarchaeota, *n* = 67), KC330823 to KC330871 (*rrs* – Euryarchaeota, *n* = 49), KC349137 to KC349317 (*amoA*, *n* = 181), and KC349318 to KC349551 (*accA*, *n* = 234).

Operational taxonomic units (OTUs) were determined from sequence alignments using mothur (v. 1.21.1; Schloss et al., 2009) with cutoffs of 0.02 (≥98% similarity) for Thaumarchaeota *rrs* and 0.03 (≥97% similarity) for Archaeal *amoA* and *accA*. Diversity indices and richness estimates (Shannon, Simpson, Chao, and ACE) were calculated in mothur. Neighbor-joining trees were constructed using ARB (Ludwig et al., 2004) with the Jukes–Cantor correction and 1,000 bootstrap resamplings for nucleotide trees; protein trees were constructed without the Kimura correction and re-sampled 100 times. Trees were edited using FigTree (v. 1.3.1)^3^.

### Pyrosequencing analyses

We also analyzed the distribution of ribotypes in 41 of our 52 samples by massively parallel sequencing (pyrosequencing) using a Roche 454/FLX instrument running Titanium chemistry. *rrs* in DNA extracted from our samples were amplified by PCR using universal *rrs* primers 515F and 806R (Table A1 in Appendix), modified for bar-coded pyrosequencing. PCR protocols and primer sequences, including barcodes, adaptors, and linkers, followed Bates et al. (2011). Purified DNA from three reactions for each sample was pooled to produce a mixture in which amplicons from each sample were represented equally. The final mixture was sequenced using standard protocols by Engencore (University of South Carolina, Columbia, SC, USA). Sequence data have been deposited with MG-RAST^4^ at accession numbers 4509220.3–4509263.3. Metadata are available via the project page: “Analysis of composition and structure of coastal to mesopelagic bacterioplankton communities in the nGoM.”

A total of 435,290 sequences were filtered and trimmed (minimum length 200 bp, minimum quality score 20; 221,410 sequences passed) and then sorted into OTUs using the PANGEA pipeline (Giongo et al., 2010). Phylogenetic affiliations of these sequences were determined by a megablast analysis using a reference set of more than 170,000 *rrs* sequences from described isolates obtained from the RDP II database (Giongo et al., 2010). Amplicon sequences were binned into OTUs at domain, phylum, class, order, family, genus, and species levels based on megablast results, and then grouped into phylogenetic clusters and sorted by station and depth (average number of sequences per sample: 5,400; range 764–9,176). The PANGEA pipeline assigns all Archaea sequences to one group that also includes divergent Bacteria sequences. In order to more accurately assess the proportion of Thaumarchaeota in our samples, we manually enumerated hits to Thaumarchaeota in the megablast output for each sample. We also counted hits to known AOB, NOB, and Euryarchaeota.

Thaumarchaeota *rrs* sequences obtained from pyrosequencing were included for phylogenetic analysis using mothur (v. 1.21.1; Schloss et al., 2009). Unique sequences were grouped together and aligned against the Silva Archaea reference database^5^. The resulting alignment, including *rrs* sequences from Station D5 clone libraries and outgroups, was trimmed to a set length and eight chimeric sequences were removed with Uchime (Edgar et al., 2011); additional potential chimeras and erroneous sequences were checked manually using BLAST and removed if necessary. The remaining 23,677 Thaumarchaeota sequences were clustered and representatives from each OTU obtained. A maximum likelihood tree was constructing using representative sequences grouped at 98% similarity (2,772 sequences total) with the RAxML program (Stamatakis et al., 2005) within ARB (Ludwig et al., 2004); 100 trees were generated using rapid bootstrap analysis, and the consensus tree was constructed from these iterations. Rarefaction analysis was completed using mothur as described for clone library samples above. The Bacteria populations of these samples are analyzed in King et al. (2013).

### Statistical analyses

Model II ordinary least squares pairwise regressions were calculated following Legendre and Legendre (1998) using software available at the R-Project web site^6^. Coefficients of determination and confidence limits of regression equations were calculated from 999 bootstrap permutations. PRIMER (v.6; Clarke and Gorley, 2006) was used to compare environmental and biological data from each station. We normalized environmental data in PRIMER to reduce the influence of variable unit scales before principal components analysis (PCA). The software package CANOCO (v. 4.5; ter Braak and Šmilauer, 2002) was used for canonical correspondence analysis (CCA; ter Braak, 1986) using PCA values and log-transformed qPCR gene abundances. Significance of CCA was determined using 499 Monte-Carlo permutations (reduced model) as recommended in the program documentation. The RAxML tree constructed from 454-generated Thaumarchaeota *rrs* sequences was used in Fast UniFrac (Hamady et al., 2009) to investigate phylogenetic patterns by sample location and depth. Weighted abundances of sequences within samples were used in both Principal Coordinates Analysis (PCoA) and sample clustering, as well as to calculate pairwise Unifrac distances. Counts were normalized to reduce the influence of larger sample sizes (greater number of sequences) at certain stations. The significance of sample clusters was tested using 100 jackknife permutations and resampling of the minimum (2), first quartile (100), or median (520) number of sequences across all samples; any sample containing less than the number of re-sampled sequences was eliminated from the analysis.

## Results

### Gene abundance and distribution

**Figure 2:**
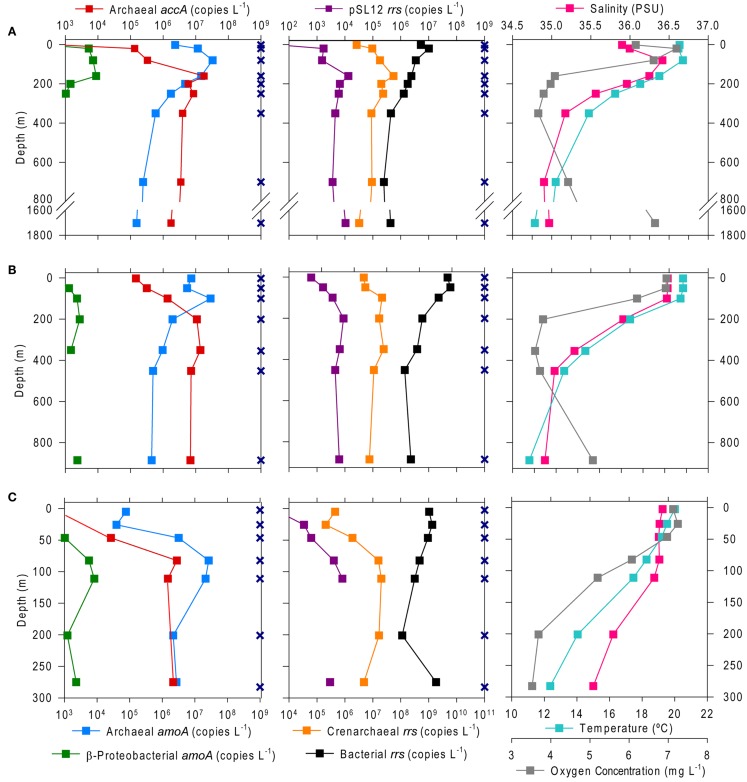
**Depth profiles of the abundance of selected genes and of environmental variables at Stations (A) A6, (B) D5, and (C) H6**. Gene abundances are given as copies L^−1^ of sample filtered as determined from triplicate qPCR amplifications of Archaeal and β-Proteobacterial *amoA* and Archaeal *accA* (left) and Thaumarchaeota, pSL12, and Bacterial *rrs* (center); note that scales for β-Proteobacterial *amoA* and pSL12 *rrs* are reduced by 10–100 to allow for visualization of variation with depth. Environmental data were taken from a CTD attached to the frame of the rosette sampler (right). Sampling depths are shown as X’s on the depth axis; missing points indicate that the measurement was below the limit of detection (see Table A1 in Appendix for detection limits).

The abundance of Bacterial *rrs* in these samples ranged from 10^5^ to 10^10^ copies L^−1^(Table 1; Table A2 in Appendix). Thaumarchaeota *rrs* genes were present in the same samples at up to 10^8^ copies L^−1^ (Table A2 in Appendix) with population maxima occurring typically between 100 and 200 m depth and at lower [O_2_] and temperature (Figure 2). The abundance of *rrs* genes attributable to the pSL12-like clade was much lower, near the limit of detection (see Table A1 in Appendix) in most samples with a maximum abundance of 10^5^ copies L^−1^(Table A2 in Appendix). Similar trends with depth for pSL12 *rrs* were observed as Thaumarchaeota *rrs*, though pSL12 *rrs* abundance was generally 100- to 10,000-fold lower (Figure 2), except in one sample (Station H1–7 m), where pSL12 *rrs* was 10% of Thaumarchaeota *rrs*. No Thaumarchaeota *rrs* were detected at the freshwater Mississippi River station (MR1-2 m) where pSL12 *rrs* was present at 10^5^ copies L^−1^ (Table A2 in Appendix).

**Table 1:** **Summary of qPCR-estimated gene abundances (copies L^−^^1^) determined for samples from the northern Gulf of Mexico**.

	Thaum. W *amoA*	Thaum. F *amoA*	Thaum. *accA*	Thaum. *rrs*	pSL12 *rrs*	AOB *amoA*	Bacteria *rrs*
Near-surface inshore	**3.86 × 10^7^** (9.74 × 10^4^ −1.74 × 10^8^)	**5.82 × 10^6^** (9.28 × 10^2^ −2.97 × 10^7^)	**1.29 × 10^6^** (9.09 × 10^2^ −1.00 × 10^7^)	**1.85 × 10^7^** (1.37 × 10^5^ −1.10 × 10^8^)	**4.77 × 10^4^** (1.12 × 10^2^ −3.30 × 10^5^)	**3.67 × 10^5^** (6.09 × 10^3^ −2.10 × 10^6^)	**3.20 × 10^9^** (3.11 × 10^5^ −1.26 × 10^10^)
Near-surface offshore	**1.16 × 10^7^** (3.91 × 10^4^ −3.29 × 10^7^)	**4.19 × 10^6^** (1.24 × 10^5^ −1.33 × 10^7^)	**5.66 × 10^5^** (3.16 × 10^2^ −2.79 × 10^6^)	**6.95 × 10^6^** (4.79 × 10^4^ −2.14 × 10^7^)	**1.30 × 10^3^** (1.89 × 10^1^ −3.98 × 10^3^)	**2.81 × 10^3^** (1.67 × 10^2^ −7.07 × 10^3^)	**7.16 × 10^8^** (3.48 × 10^8^ −1.34 × 10^9^)
Deep offshore	**3.68 × 10^6^** (4.65 × 10^3^ −2.12 × 10^7^)	**1.11 × 10^7^** (5.11 × 10^5^ −5.86 × 10^7^)	**8.72 × 10^6^** (1.48 × 10^5^ −1.80 × 10^7^)	**1.79 × 10^7^** (3.23 × 10^6^ −5.45 × 10^7^)	**1.00 × 10^4^** (3.52 × 10^3^ −2.92 × 10^4^)	**2.93 × 10^3^** (1.34 × 10^2^ −8.80 × 10^3^)	**2.14 × 10^8^** (2.49 × 10^7^ −1.83 × 10^9^)

*Means for each reaction are listed in bold; ranges follow the mean in parentheses. *amoA* W, amplified with Wuchter et al. (2006) *amoA* primer set; *amoA* F, amplified with Francis et al. (2005) *amoA* primer set. “Near-surface” is ≤100 m depth; “deep” is >100 m depth; “inshore,” over the continental shelf (seafloor depth < 100 m); “offshore,” shelf break and beyond (depth > 100 m)*.

**Figure 3:**
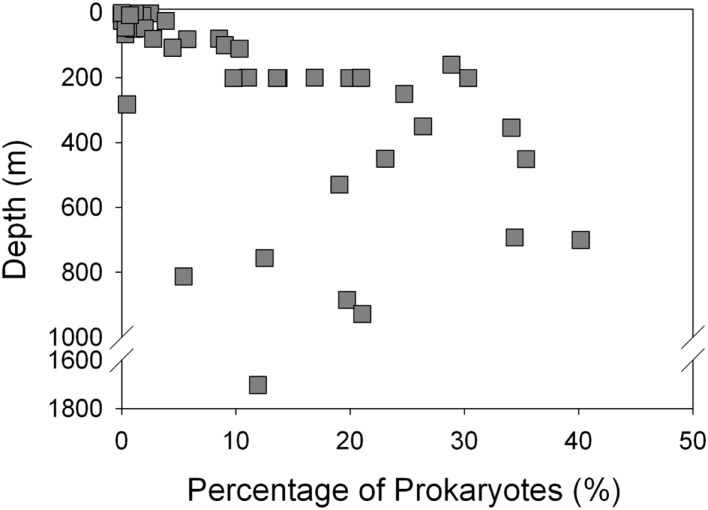
**Abundance of Thaumarchaeota as a percentage of total bacterioplankton plotted against sample depth**.

**Figure 4:**
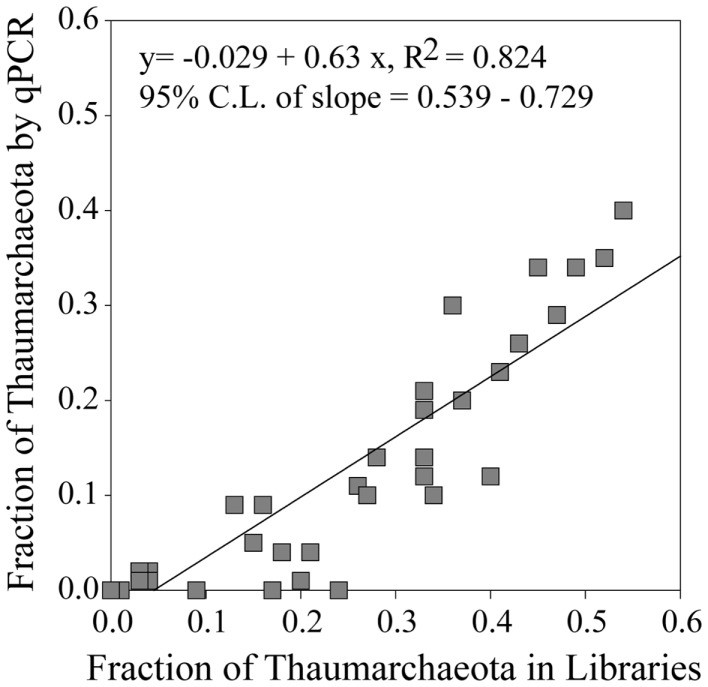
**Fraction of Thaumarchaeota *rrs* found in 454 pyrosequencing libraries versus the fraction of Thaumarchaeota *rrs* determined from qPCR data**. Line represents a model II pairwise regression.

Thaumarchaeota accounted for a high proportion (up to 40% by qPCR, up to 54% of pyrosequenced *rrs*) of the total prokaryotic community in our samples. This percentage varied with depth (Figure 3), with deeper (>100 m) samples containing an average of 21% Thaumarchaeota (range 0.5–40%) while samples from near-surface water (≤100 m) contained only 1.8% Thaumarchaeota (range 0–9%). Differences were also observed with distance from shore, with shallower (<100 m) samples from inshore stations having fewer Thaumarchaeota than those from offshore stations (1.1 versus 2.8% of prokaryotes, respectively). Pyrosequencing also showed that Thaumarchaeota *rrs* genes were most abundant in samples from depths of 100–200 m, though they were present at low abundances in all samples with the exception of MR1-2 m (Table A3 in Appendix), in agreement with qPCR analyses. Thaumarchaeota accounted for 0.1–54% of the prokaryotes in pyrosequencing libraries and their distributions based on qPCR estimates of gene abundance compared favorably with the contribution of Thaumarchaeota ribotypes to pyrosequenced *rrs* libraries from these samples (Figure 4; model II regression, *n* = 41, *r*^2^ = 0.82, 95% CL of slope = 0.54–0.73).

Archaeal *amoA* was present at up to 10^8^ copies L^−1^ (Table 1; Figure 2; Table A2 in Appendix). Bacterial *amoA* was at the limit of detection (Table A1 in Appendix) in most samples, with a maximum of 10^6^ copies L^−1^. The ratio of AOA:AOB *amoA* was found on average to be 2100:1 (Wuchter primers) to 3300:1 (Francis primers). The ratio of Bacterial *amoA*:Bacterial *rrs* averaged 0.001 across all samples, with a maximum of 0.05 at Station D3–68 m (Figure A5A in Appendix). Abundances of *accA* genes ranged from the limit of detection (10^4^ copies L^−1^) to 10^7^ copies L^−1^(Table 1; Figure 2; Table A2 in Appendix). Archaeal *amoA* (quantified using Wuchter primers) showed similar distribution by depth as Thaumarchaeota *rrs* (Figure 2). However, *accA* abundances showed opposite trends with depth, leading to higher ratios of *amoA*:*accA* or *rrs*:*accA* in near-surface (≤100 m) water (Figure 2; Table 2).

**Table 2:** **Mean and ranges of the ratios of Thaumarchaeota gene abundances**.

	*amoA* W:*rrs*	*amoA* F:*rrs*	*accA*:*rrs*
Near-surface inshore	2.5 (0.71−6.6)	0.32 (0.002−0.69)	0.06 (0.001−0.22)
Near-surface offshore	1.2 (0.17−1.8)	0.62 (0.28−1.9)	0.04 (0.0002−0.17)
Deep offshore	0.19 (0.001−1.0)	0.57 (0.16−1.1)	0.58 (0.07−1.3)

*Gene ratios were calculated by dividing the abundance of each of the genes tested by the abundance of *rrs* in the same sample. *amoA* W, amplified with Wuchter et al. (2006) *amoA* primer set; *amoA* F, amplified with Francis et al. (2005) *amoA* primer set*.

We used PCA (Figure A3 in Appendix) to identify samples from similar environments and group them into a few categories to simplify comparisons. The first two PCA axes explained 63.2% of the variation between samples (Figure A3; Table A5 in Appendix), which supported placing stations into three groups: near-surface inshore, near-surface offshore, and deep offshore sets. CCA was included (Figure 8) to investigate relationships between gene abundances and environmental conditions (similar to BEST analysis, see Appendix). The primary CCA axis (CCA1) explained 47.9% of the gene abundance-environment relationship; adding the second axis (CCA2) increased the variance explained by 44% (91.7% total; Figure 8; Table A6 in Appendix). A global permutation test gave a statistical significance of *p* < 0.05 for station groupings based on both canonical axes considered together (*F* = 2.26, *p* = 0.014), while CCA1 considered alone did not explain the gene abundance-environment relationship (*F* = 8.43, *p* = 0.086). Thaumarchaeota *rrs* abundance was negatively correlated with most environmental variables, except for salinity and depth (Figure 8). Bacterial *rrs* abundance correlated positively with euphotic zone depth and had a strong negative correlation with pH, with little influence from any variable primarily contributing to CCA2 (beam attenuation, oxygen; Figure 8). The distribution of Archaeal *amoA* genes as assessed with the Wuchter primers, in contrast, was not strongly influenced by variables contributing to CCA1 (fluorescence, pH, latitude, longitude; Figure 8) but showed a weak positive correlation with temperature and beam attenuation (turbidity). Archaeal *amoA* gene abundance assessed by the Francis primers showed the opposite trend, with strongest positive correlations to latitude (which covaries with distance offshore and depth in this region) and oxygen concentrations (Figure 8). Bacterial *amoA* gene abundance correlated with beam attenuation (turbidity) and temperature (positive correlation), as well as depth (negative correlation). *accA* gene abundance had strong positive correlations with relative fluorescence (chlorophyll a equivalents) and pH (Figure 8).

### Thaumarchaeota community composition at station D5

**Figure 5:**
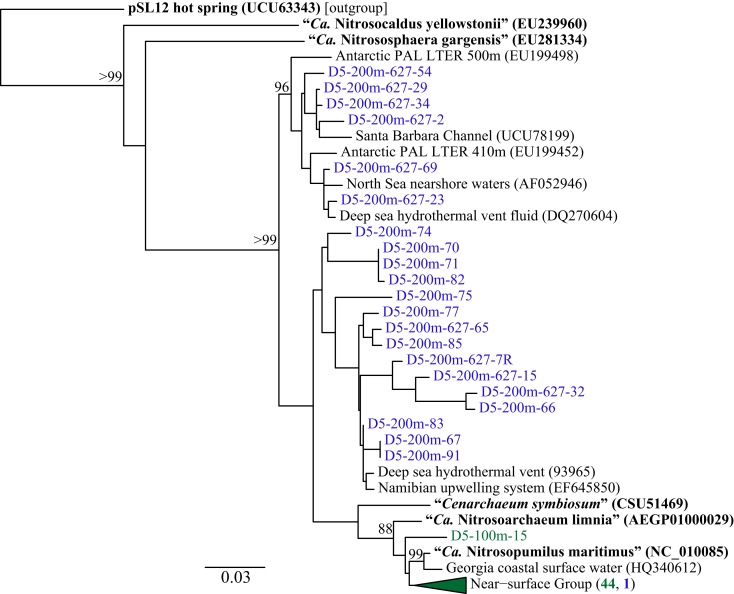
**Phylogenetic analysis of Thaumarchaeota *rrs* genes retrieved from Station D5**. Clone libraries were generated from DNA in samples collected at depths of 100 m (green) and 200 m (blue). The Neighbor-Joining tree was constructed using ARB (Ludwig et al., 2004). Reference sequences in bold are from isolates or enrichment cultures of AOA. Bootstrap values obtained from resampling 1000 times; only values above 75% bootstrap support are shown.

Phylogenetic analysis of 67 Sanger-sequenced Thaumarchaeota *rrs* sequences obtained from 100 and 200 m depth at Station D5 revealed 10 different OTUs (Figure 5; Table A4 in Appendix; 98% similarity cutoff). All but one of the sequences retrieved from the 100 m sample clustered into a single OTU (the “Near-Surface Group,” Figure 5), that also contained one sequence retrieved from the 200 m sample and the reference sequence from *Nitrosopumilus* sp. NM25 (AB546961; Matsutani et al., 2011). We did not retrieve any sequences related to the marine pSL12-like clade. Sequences retrieved from the 200 m sample displayed greater richness and evenness (Table A4 in Appendix; 9 OTUs) and included some OTUs that appear unique to the northern Gulf of Mexico.

**Figure 6:**
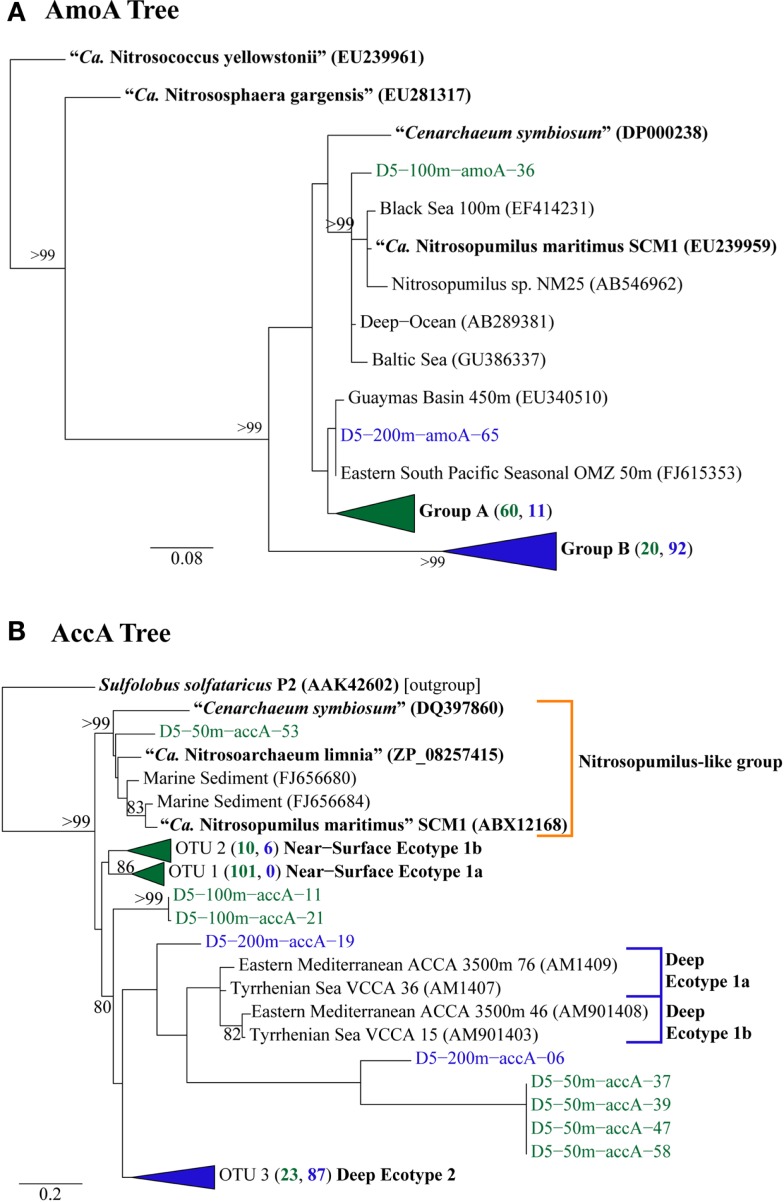
**Phylogenetic analysis of inferred amino acid sequences from (A) *amoA* and (B) *accA* gene sequences retrieved from Station D5**. Numbers beside groups (in triangles) indicate the number of sequences from each depth sampled according to color: clades in green are from 2, 50, or 100 m; clades in blue are from 200 or 450 m. Neighbor-Joining Trees were constructed with ARB (Ludwig et al., 2004) from sequences 199 aa (AmoA) or 137 aa (AccA) in length. Sequences in bold were obtained from isolates or enrichment cultures of AOA. Bootstrap values were obtained from 100 resamplings; only values above 75% bootstrap support are shown.

**Figure A1:**
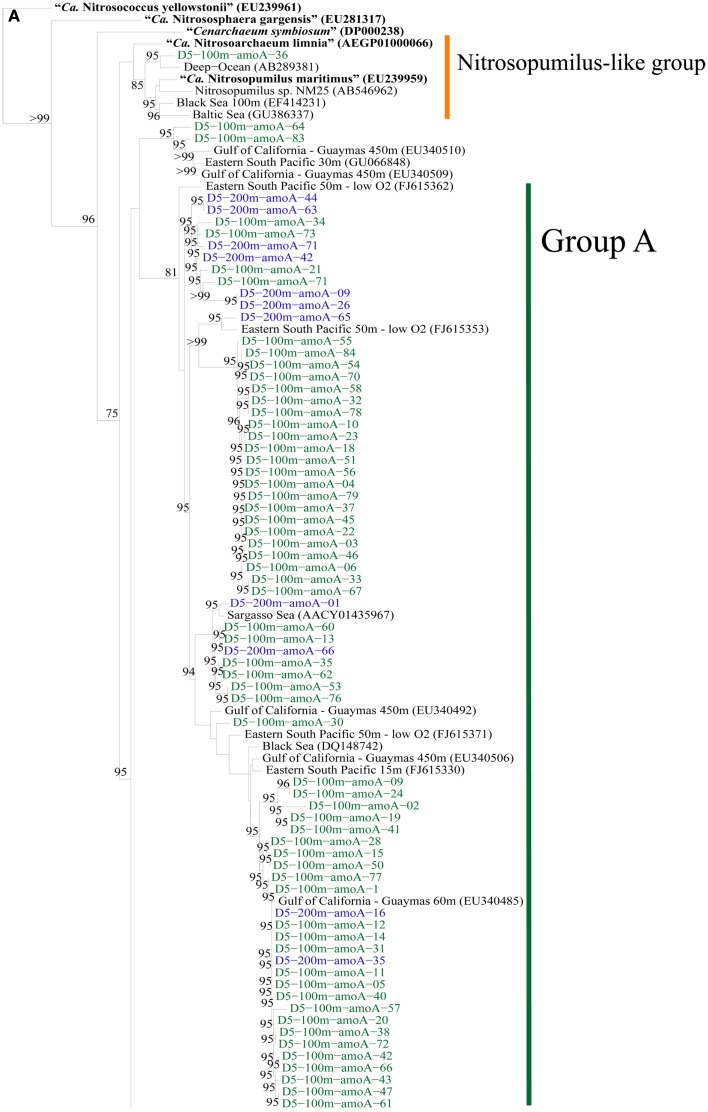

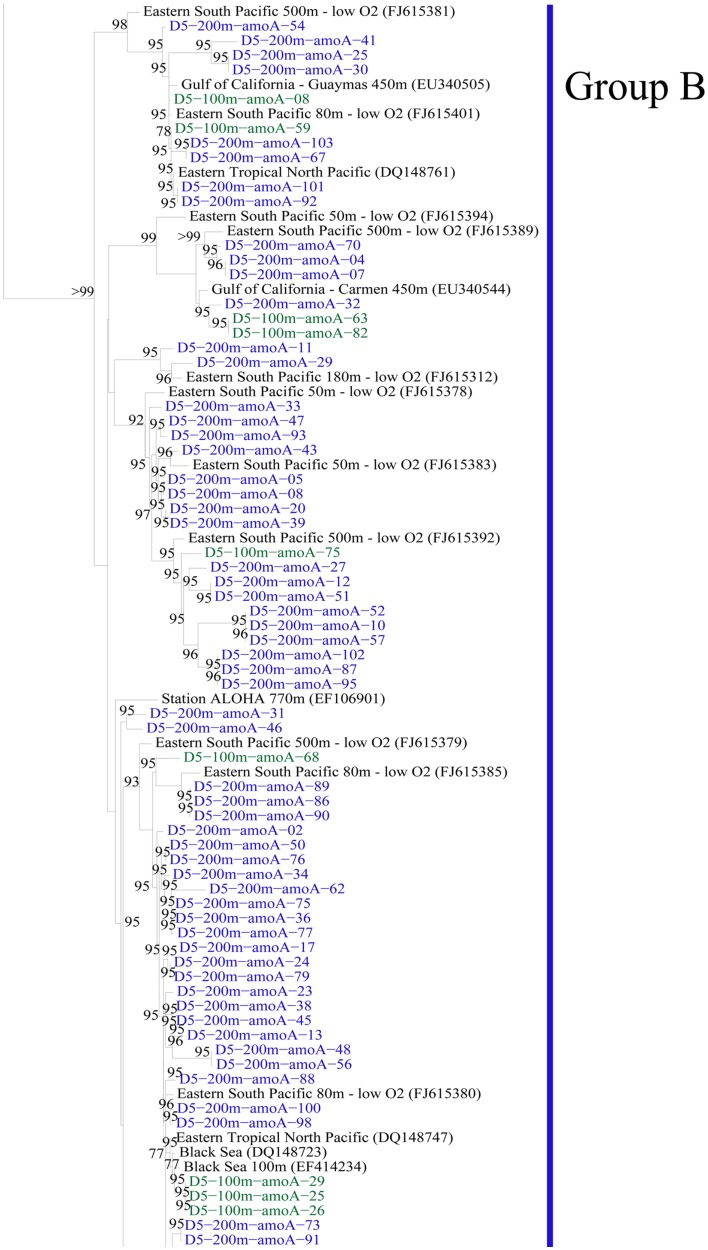

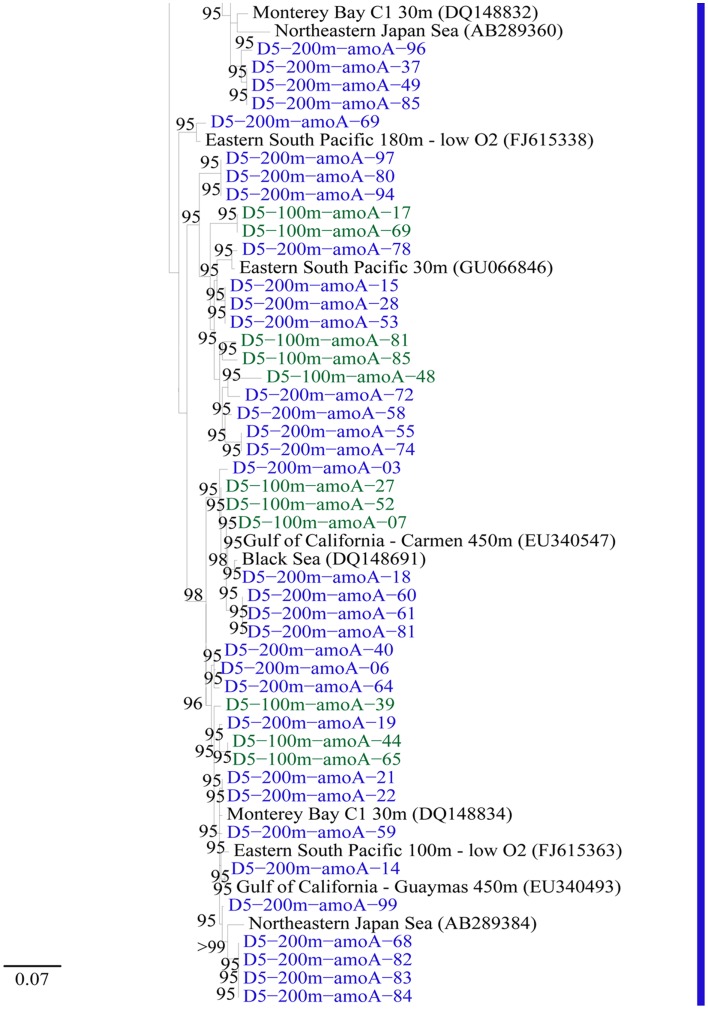

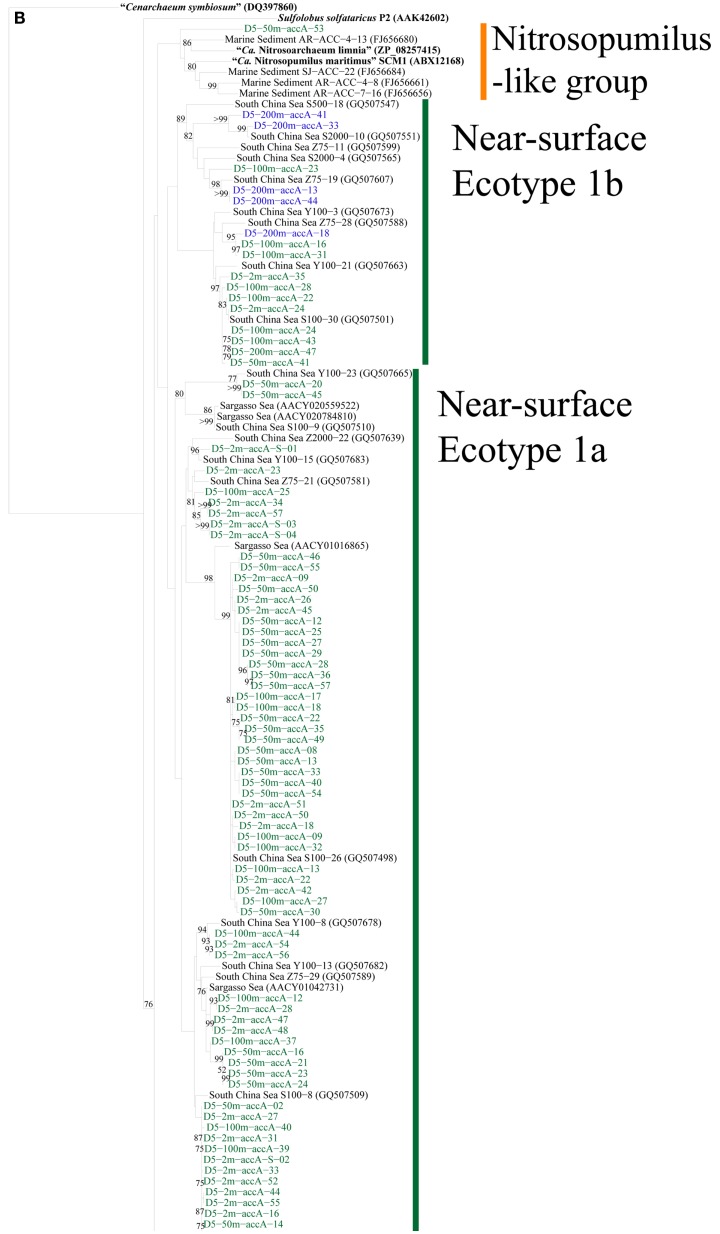

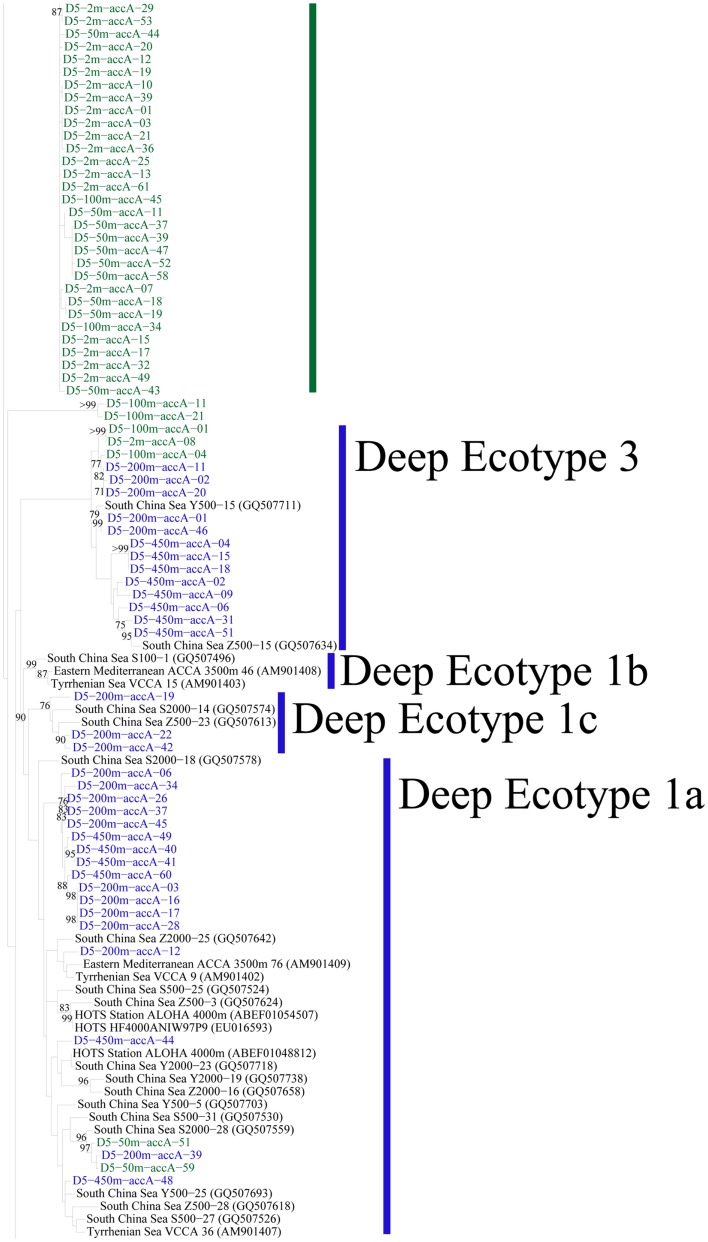

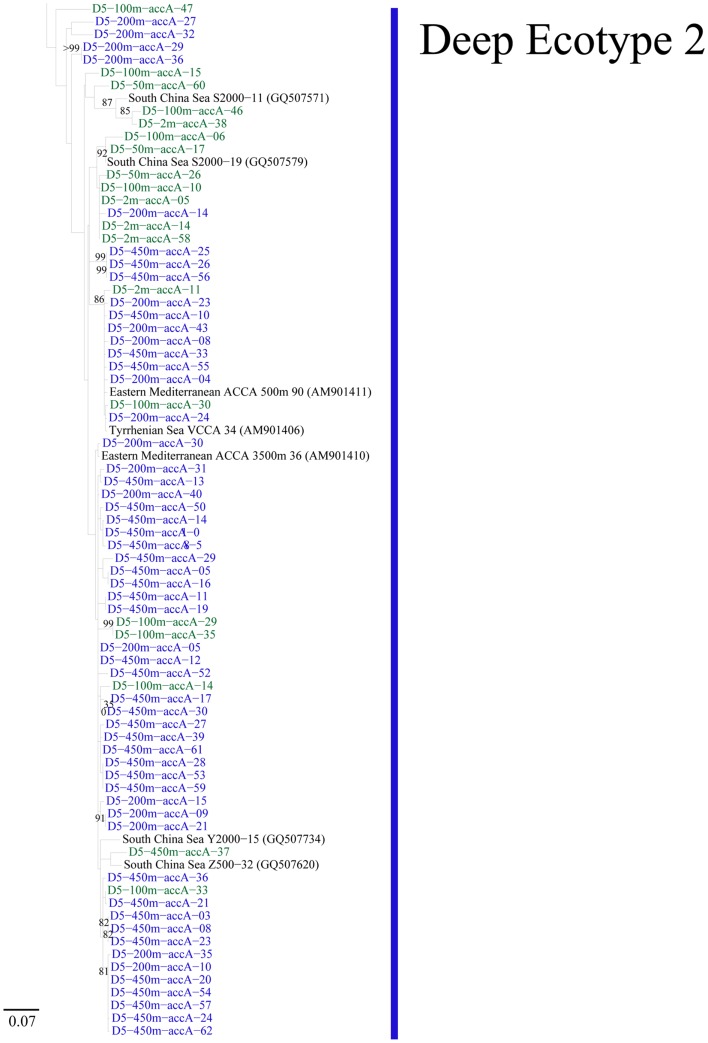
**Phylogenetic analysis of (A) *amoA* and (B) *accA* genes retrieved from Station D5**. Neighbor-Joining Trees built with ARB (Ludwig et al., 2004) from nucleotide sequences 595 bp (*amoA*) or 411 bp (*accA*) in length. Sequences in bold obtained from isolates or enrichment cultures. Bootstrap values obtained from resampling tree 1,000 times; only values above 75% bootstrap support shown on tree.

We retrieved 184 *amoA* sequences from Station D5. Phylogenetic analysis of the translated and aligned amino acid sequences revealed two OTUs (similarity cutoff of 97%) of AmoA (Figure 6A): one containing primarily near-surface (100 m) sequences (“Group A” following Beman et al., 2008) and the other dominated by sequences from 200 m (“Group B”). *amoA* nucleotide sequences also grouped primarily by depth, but with greater richness and diversity (Table A4 in Appendix) at a given depth than we observed for Thaumarchaeota *rrs* genes. Clusters of sequences that appear to be unique to the Gulf of Mexico were observed in both 100 and 200 m samples (Figure A1A in Appendix).

The top BLASTx hits for all but 30 of 257 sequences obtained from *accA* amplicons were to carboxylase or carboxyltransferase genes from Archaea. The remaining 30 amplicons were most similar to non-Thaumarchaeota reference sequences with low (≤65%) sequence identities. Because they did not return hits to Thaumarchaeota reference sequences, we did not consider them further. Phylogenetic analysis of the inferred amino acid sequences for AccA (Figure 6B) revealed three major OTUs: OTU 1 contained a majority of near-surface sequences (2, 50, and 100 m), while OTUs 2 and 3 contained mostly sequences from deep water (200 and 450 m). Analysis of *accA* nucleotide sequences revealed similar clusters with depth as inferred amino acid sequences for AccA and Thaumarchaeota *rrs* gene sequences (Figure A1B in Appendix) with a total of 51 OTUs observed at a 97% similarity cutoff (Table A4 in Appendix). Some of these seem unique to the Gulf of Mexico (Figure A1B in Appendix), but this may be an artifact of the limited representation of *accA* sequences in reference databases.

### Pyrosequencing: Phylogenetic patterns and sample groupings

**Figure A2:**
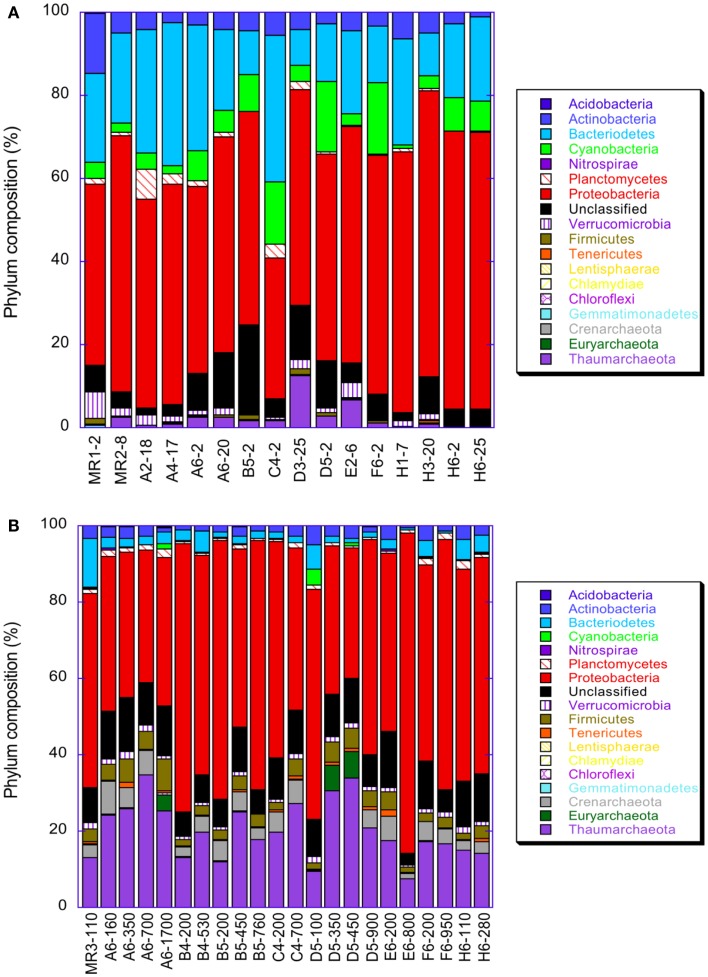
**Distribution of various taxa in pyrosequenced libraries of *rrs* in samples from the northern Gulf of Mexico: (A) samples taken from depths ≤25 m; (B) samples from depths ≥100 m**.

Microbial community composition varied dramatically with depth as shown by comparisons of libraries from surface (≤25 m depth) versus subsurface (≥100 m depth) samples (Figure A2 in Appendix, Table A3 in Appendix; these data are discussed fully in King et al., 2013). Proteobacteria, especially α- and γ-Proteobacteria, dominated the microbial community of near-surface waters at most stations. Consistent with distributions of *rrs* and *amoA* indicated by qPCR analyses, Thaumarchaeota were greatly enriched in deeper waters. Only 14 (out of a total of 221,410) *rrs* sequences binned to AOB, confirming the much lower abundance of AOB relative to AOA found by qPCR quantification of *amoA*. Half of the AOB sequences were retrieved from one sample: MR1–2 m, taken upstream of the mouth of the Mississippi River with a salinity of 0. Only four Thaumarchaeota sequences were retrieved from this sample (Table A3 in Appendix), two of which were most similar to the terrestrial thaumarchaeota, “*Candidatus* *Nitrososphaera gargensis*” strain EN76, at 15% similarity.

**Figure 7:**
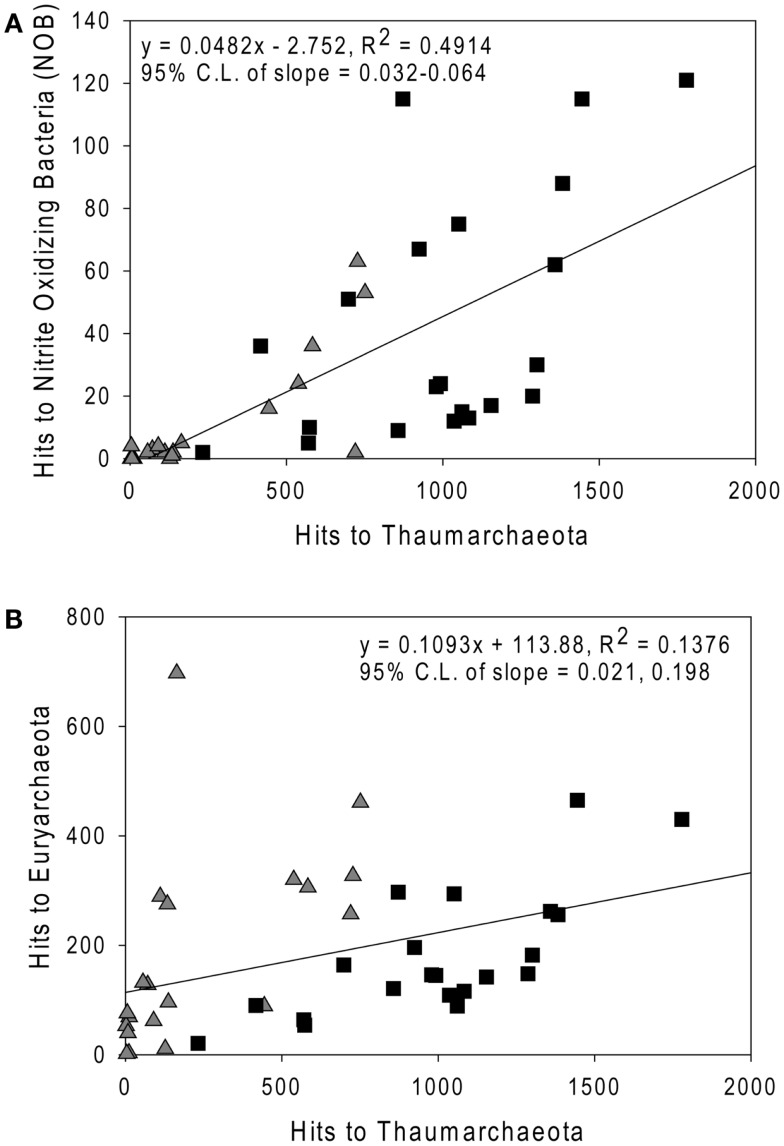
**Comparison of the abundance of *rrs* from (A) Nitrite-Oxidizing Bacteria; and (B) Euryarchaeota versus Thaumarchaeota *rrs* in samples from the northern Gulf of Mexico**. Triangles, near-surface (≤100 m) samples; squares, Deep (>100 m) samples. Lines are model II regressions (Legendre and Legendre, 1998) of all data.

Sequences most closely related to NOB were retrieved from most samples (mean = 0.4%, range 0–1.8% of prokaryotes as calculated in “Methods” in Appendix, but assuming 2 *rrs* per NOB genome from Mincer et al., 2007). These sequences were primarily identified as *Nitrospina* sp. 3005 (AM110965), though *Nitrospira* ribotypes were also detected. The abundance of NOB *rrs* was greatest at depth (∼200 m, Table A3 in Appendix, Figure 7A) and was significantly correlated with the abundance of Thaumarchaeota in the same samples (Figure 7A; model II regression, *n* = 41, *r*^2^ = 0.49, 95% CL of slope = 0.032–0.064). Euryarchaeota only accounted for a few percent of the microbial community (mean 5.8%, range 0.1–17.6%). Euryarchaeota were most abundant in near-surface samples (<100 m; Table A3 in Appendix) and their abundance was poorly correlated with the abundance of Thaumarchaeota (Figure 7B; model II regression, *n* = 41, *r*^2^ = 0.14, 95% CL of slope = 0.021–0.20).

UniFrac distances calculated between samples indicate significant (*p* ≤ 0.05) similarities in Thaumarchaeota *rrs* assemblages among offshore, near-surface samples and inshore, near-surface samples from Stations A2, A4, D3, E2, and MR2 (data not shown). The Station D5–100 m sample was assigned to the near-surface group (*p* ≤ 0.05) regardless of the method used to obtain *rrs* sequences (pyrosequencing versus Sanger sequencing from clone libraries). Among deep offshore samples, those from 160–950 m were similar to each other (*p* ≤ 0.05); sequences from clone libraries generated from Station D5–200 m were also included in this group. The phylogenetic composition of Thaumarchaeota *rrs* in the deepest sample, Station A6–1700 m, was only similar to samples from D5–900 m and F6–950 m (*p* ≤ 0.05).

**Figure A3:**
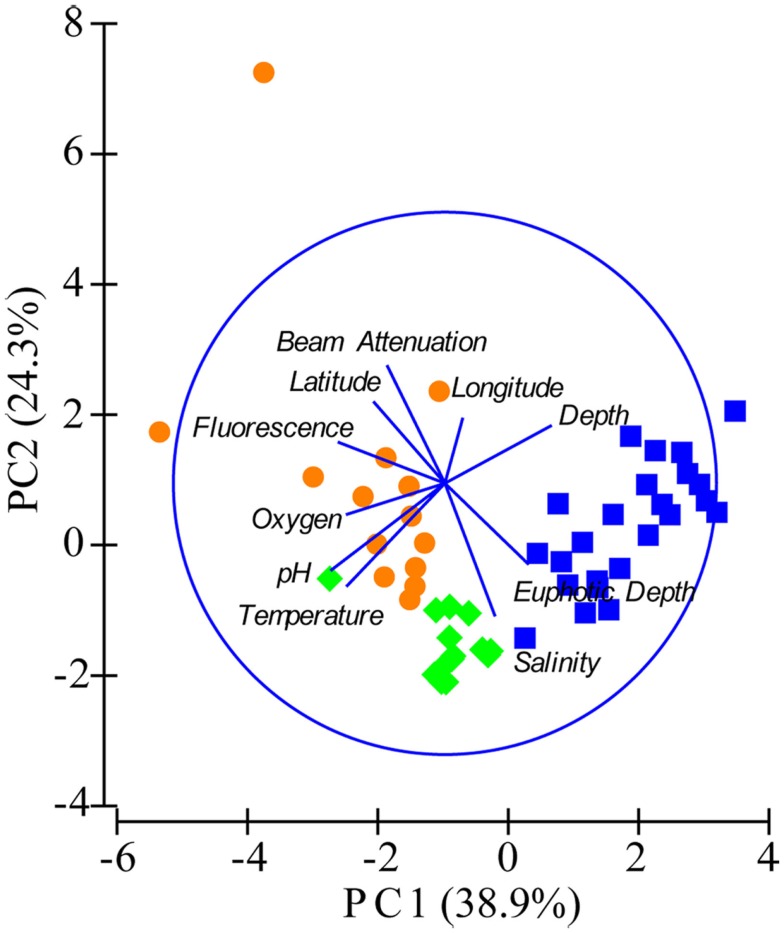
**Principal components analysis (PCA) of samples using environmental variables**. Fluorescence, relative fluorescence, chlorophylla equivalents; beam attenuation, turbidity. Samples are shown as symbols representing three groupings based on depth and location: orange circles = near-surface inshore (≤100 m depth, over the continental shelf), green diamonds = near-surface offshore (≤100 m depth, shelf break and beyond), and blue squares = deep offshore (>100 m, shelf break and beyond).

**Figure A4:**
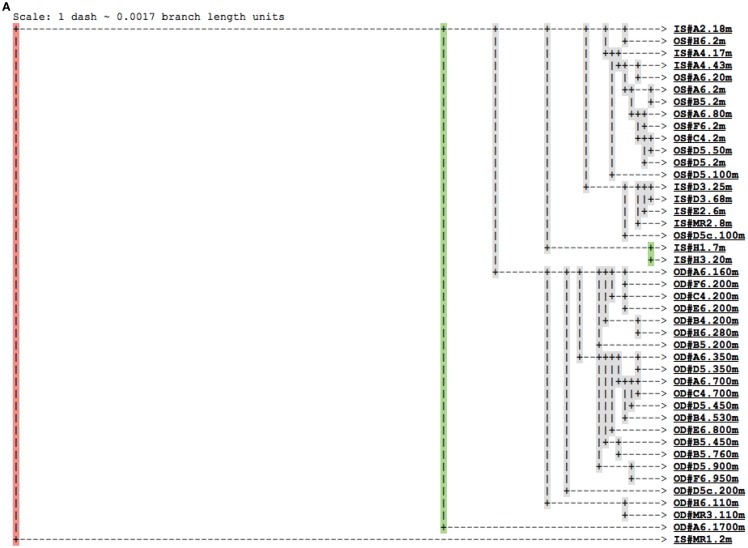

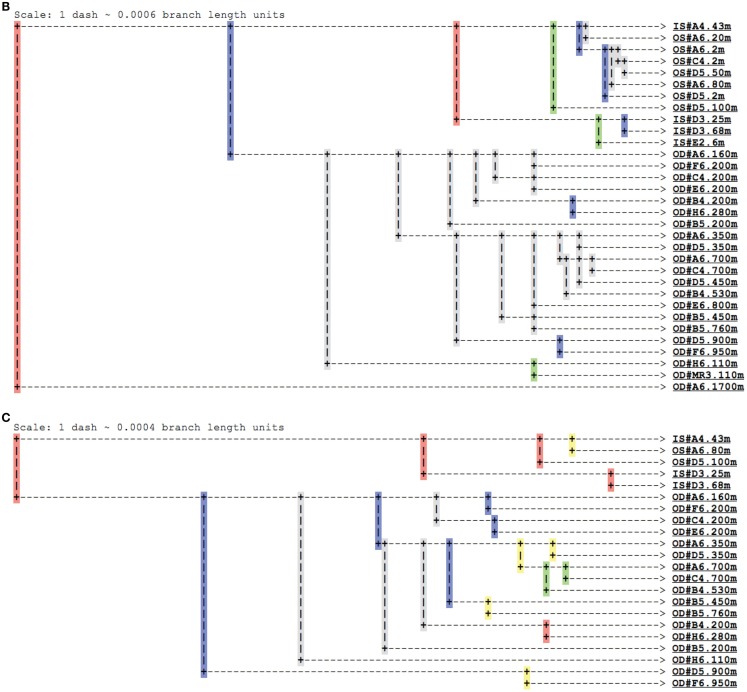
**Jackknife clustering analysis of pyrosequenced Thaumarchaeota rrs genes using Fast UniFrac (Hamady et al., 2009)**. Resampling of **(A)** 2 (minimum; *n* = 43 samples), **(B)** 100 (first quartile; *n* = 32 samples), or **(C)** 520 (median; *n* = 22 samples) sequences were performed for each of 100 iterations of the jackknife analysis. Colors indicate the percentage of iterations supporting a given node – red (>99.9%), yellow (90–99.9%), green (70–90%), blue (50–70%), or gray (<50%). Sample groups are indicated as: IS# = inshore, near-surface; OS# = offshore, near-surface; OD# = offshore, deep; with # indicating the sample as Station.Depth.

**Figure A5:**
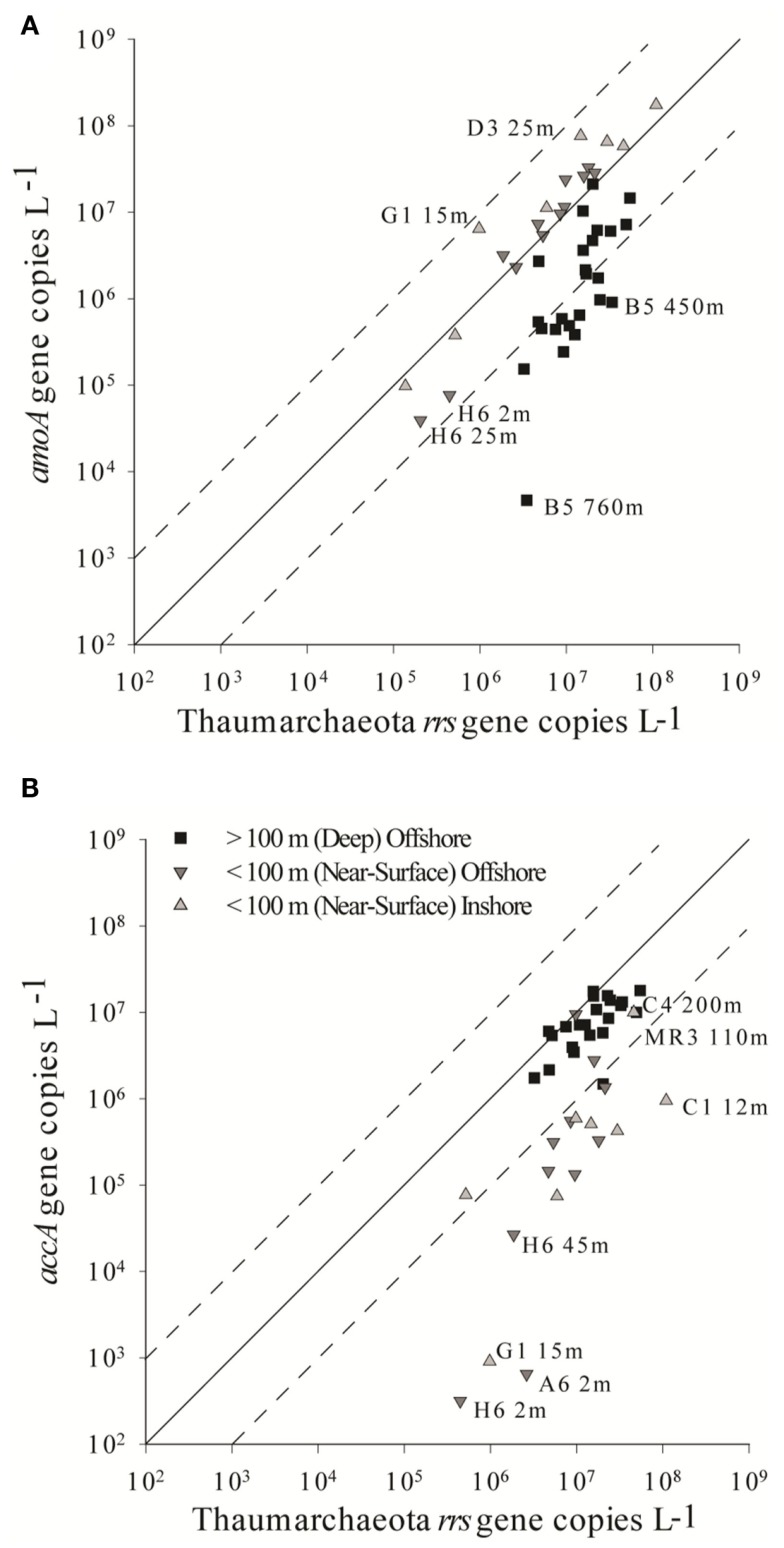
**Scatter plot of Archaeal (A) *amoA* and (B) *accA* versus *rrs* gene abundance in samples from the northern Gulf of Mexico**. Solid lines indicate the 1:1 ratios expected from the “*Ca. N. maritimus*” strain SCM1 genome (Walker et al., 2010); dashed lines indicate ratios of 0.1 or 10. “Inshore,” over the continental shelf; “offshore,” shelf break and beyond.

Analysis of phylogenetic patterns across samples using PCoA in Fast UniFrac (Figure 9) revealed two major groups of pyrosequenced Thaumarchaeota *rrs* – one of deep (>100 m) samples and another including the near-surface samples (both inshore and offshore), which agrees with PCA groupings (Figure A3 in Appendix). The primary PCoA axis explained 70% of the variation in phylogenetic composition of the samples, with the secondary axis explaining an additional 11% (total 81%) of the variation. The sample from Mississippi River Station MR1 was an outlier; however, PCoA analysis with this sample included revealed the same general pattern (Figure A7 in Appendix). Samples clustered using the minimum resampling of 2 sequences (Figure A4A in Appendix) only showed significant separation of Station MR1 sample from the rest of the samples (>99.9% jackknife support). For 100 re-sampled sequences (32 of 43 samples; Figure A4B in Appendix), a clear separation was observed between surface and deep samples (60% support) and between near-surface inshore samples (excluding Station A4) and near-surface offshore samples (>99.9% support). When the median number of sequences was applied to cluster analysis (520 sequences, 22 of 43 samples; Figure A4C in Appendix), the separation of deep and near-surface samples was statistically significant (>99.9% support). Station D3 (inshore, <100 m depth) samples clustered most closely (>99.9% support), followed by inshore Station A4–43 m and offshore Station A6–80 m (95% support). Amongst deep samples, a further separation was observed within the deep offshore samples, with the deepest samples (Stations D5–900 m and F6–950 m) and those from 350–760 m forming distinct clusters 50 and 61% of the time, respectively (Figure A4C in Appendix).

## Discussion

### Community comparisons

We found a strong correlation between qPCR and pyrosequencing estimates of AOA relative abundance indicating that, despite potential biases associated with individual qPCR primers, qPCR estimates of Thaumarchaeota distributions at this coastal site are robust. Thaumarchaeota were abundant in deeper waters of the northern Gulf of Mexico, increasing in abundance with depth to a broad maximum between ∼200 and 800 m (Figures 2 and 3), coinciding with the oxygen minimum (Figure 2). Two shallow water stations (C1, 12 m; MR2, 8 m) contained up to 10^8^ copies L^−1^ of Thaumarchaeota *rrs*; both of these stations are near the Mississippi River Plume, which may indicate an influence of riverine nutrients on AOA. It is important to note, however, that these are marine ribotypes and not terrestrial or freshwater ribotypes carried into the Gulf by the Mississippi River, since we did not retrieve similar ribotypes from Mississippi River sample MR1. In contrast, AOB *amoA* genes were below the limit of detection except in a few near-surface samples from inshore stations (Stations C1, D3, D5, G1, and H1) and in river stations MR1 and MR2. Consistent with many other studies of *amoA* in coastal water columns (Wuchter et al., 2006; Herfort et al., 2007; Beman et al., 2010), AOA *amoA* was always >10- to 100-fold more abundant than AOB *amoA*. The relative abundance of Thaumarchaeota and AOB *rrs* in pyrosequenced libraries (Table A3 in Appendix) is consistent with the distribution of *amoA* genes determined by qPCR, suggesting that the observed ratio of AOA:AOB *amoA* is not an artifact of primer bias. Although we do not have ammonia oxidation rate measurements for these samples, the greater abundance of AOA than AOB *amoA* suggests that Thaumarchaeota are likely to dominate nitrification in this region (Beman et al., 2008).

We did not quantify the distribution of NOB by qPCR (cf. Santoro et al., 2010, which is limited to *Nitrospina*); however, we were able to determine the distribution of all known NOB relative to Thaumarchaeota from pyrosequenced *rrs* libraries. We found that NOB abundance correlated well with that of Thaumarchaeota (*r*^2^ = 0.49), as reported by others (Mincer et al., 2007; Santoro et al., 2010). The correlation between the distributions of these two groups suggests relatively tight coupling between them, presumably leading to efficient conversion of ammonia to nitrate in the northern Gulf of Mexico. However, NOB *rrs* abundance was only ∼5% of that of Thaumarchaeota (slope of model II regression; Figure 7A), in contrast to estimates of 20–100% reported by Mincer et al. (2007) or ∼25% reported by Santoro et al. (2010). This ratio would change if the *rrs* gene dosages we used in our calculations changed; however, the discrepancy suggests that alternative pathways, e.g., anammox, might be more significant for nitrite removal in the northern Gulf of Mexico than in the temperate Pacific upwelling zone sampled by Mincer et al. (2007) and Santoro et al. (2010).

### Environmental factors

**Figure 8:**
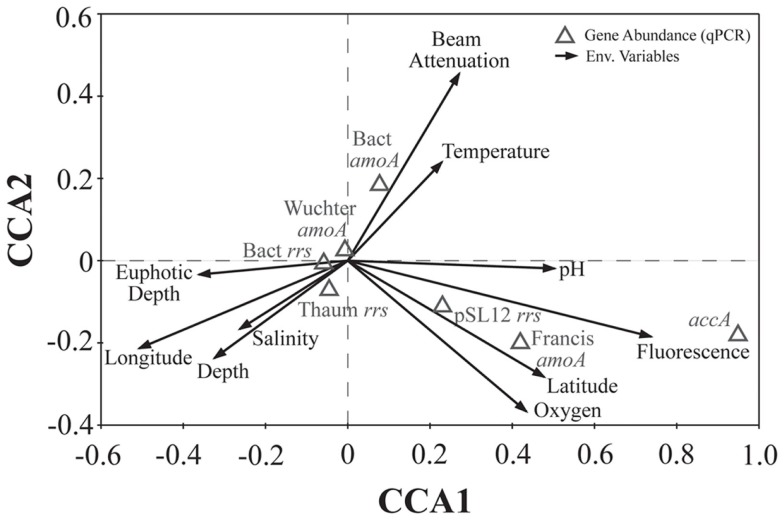
**Canonical correspondence analysis (CCA) ordination plot of qPCR-estimated abundances for *rrs*, *amoA*, and *accA* genes and environmental data**. The length and angle of arrows shows the contribution of a particular environmental variable to the CCA axes. Fluorescence, relative fluorescence, chlorophyll a equivalents; beam attenuation, turbidity. Eigenvalues, correlation values, and percentage variance for CCA are given in Table A6 in Appendix.

The connection between pH and AOA abundance has been examined closely in soils, where Archaeal *amoA* typically dominates in more acidic samples (reviewed in Prosser and Nicol, 2008; Erguder et al., 2009). The Mississippi River plume is a site of respiration-induced acidification (Cai et al., 2011), and we observed a negative correlation between the abundance of Thaumarchaeota *rrs* and pH in our samples. In contrast, the abundance of Archaeal *accA* genes and of AOA *amoA* genes detected by the Francis primers was positively correlated with pH values (Figure 8). AOB *amoA* abundance was positively correlated with temperature and negatively correlated with depth, while AOA *amoA* abundance showed the opposite trends (Figure 8). These correlations correspond to AOB abundance being greatest in surface samples, versus AOA abundance being greater in samples from deeper, colder water, as observed in other studies (e.g., Santoro et al., 2010). We also observed a strong negative correlation between AOB *amoA* gene abundances and salinity, but we did not find a statistically significant (*p* > 0.05) correlation between AOA *amoA* genes and salinity. This contrasts with AOA distributions reported for sediments from an aquifer at Huntington Beach, CA, USA (Santoro et al., 2008) or from the San Francisco Bay Estuary (Mosier and Francis, 2008), where AOB were more abundant in high salinity sediments, while AOA were more prominent in low salinity environments.

Fluorescence (chlorophyll a) contributed significantly to PC1 (Figure A3 in Appendix) and *accA*, pSL12 *rrs*, and Archaeal *amoA* gene abundance (Francis primers) were all positively correlated with fluorescence in CCA analysis (Figure 8). Most other studies have reported inverse correlations between Thaumarchaeota abundance and chlorophyll a (Murray et al., 1999a,b; Wells and Deming, 2003; Kirchman et al., 2007). A study of AOA and AOB dynamics in estuarine sediments, though, showed that potential nitrification rates and the abundance of Archaeal *amoA* genes (Wuchter primers) correlated positively with sediment chlorophyll a concentrations (Caffrey et al., 2007). Archaeal abundance in the Arctic Ocean near the Mackenzie River mouth correlated positively with chlorophyll a (Wells et al., 2006), although a previous study at similar sites showed the opposite trend (Wells and Deming, 2003). We observed a strong positive correlation between Bacterial *amoA* abundance and turbidity in the Gulf of Mexico while Archaeal *amoA* genes were inversely correlated with turbidity (Figure 8). We detected greatest abundances of AOB *amoA* genes in shallow, near-shore waters (especially at Station C1 and all three Mississippi River stations), which may indicate a salinity effect or an association of AOB with particles originating from estuaries, coastal embayments, or the river. Since we did not sequence the AOB amplicons we obtained, we cannot use the phylogenetic position of the AOB to differentiate between these hypotheses (e.g., Phillips et al., 1999; O’Mullan and Ward, 2005). Caffrey et al. (2007) reported that AOB were more abundant than AOA in sediments from Weeks Bay, Alabama, a subembayment of Mobile Bay. Our near-shore waters also had higher ammonia concentrations (up to 3 μM; data not shown) than at other stations, which is consistent with the conceptual model that AOB are more competitive in environments with elevated ammonia concentrations (Martens-Habbena et al., 2009).

Oxygen concentrations are typically higher in surface than deep water, especially in this region of the Gulf of Mexico where bottom waters become seasonally hypoxic (Rabalais et al., 2002, 2010). Although samples for this study were collected before hypoxia had fully developed ([O_2_] ranged from 3.5 to 8.4 mg L^−1^; 150–375 μM), we found clades of AOA similar to those observed in other hypoxic waters (Beman et al., 2008; Labrenz et al., 2010; Molina et al., 2010). Additionally, we determined that the distribution of *amoA* phylotypes detected by the Francis primers correlated positively with [O_2_] (as did Archaeal *accA* genes), while those detected by the Wuchter primers were not correlated with [O_2_] (Figure 8). Our data suggests that these primer sets have different PCR biases such that certain AOA ecotypes are amplified more efficiently by one set than the other. As we observed correlations between different environmental variables and *amoA* phylotypes amplified by each primer, we believe these differences may reflect ecotype-specific sequence variation, as proposed for the two primer sets given in Beman et al. (2008).

### *amoA* and *accA* abundance

**Figure A6:**
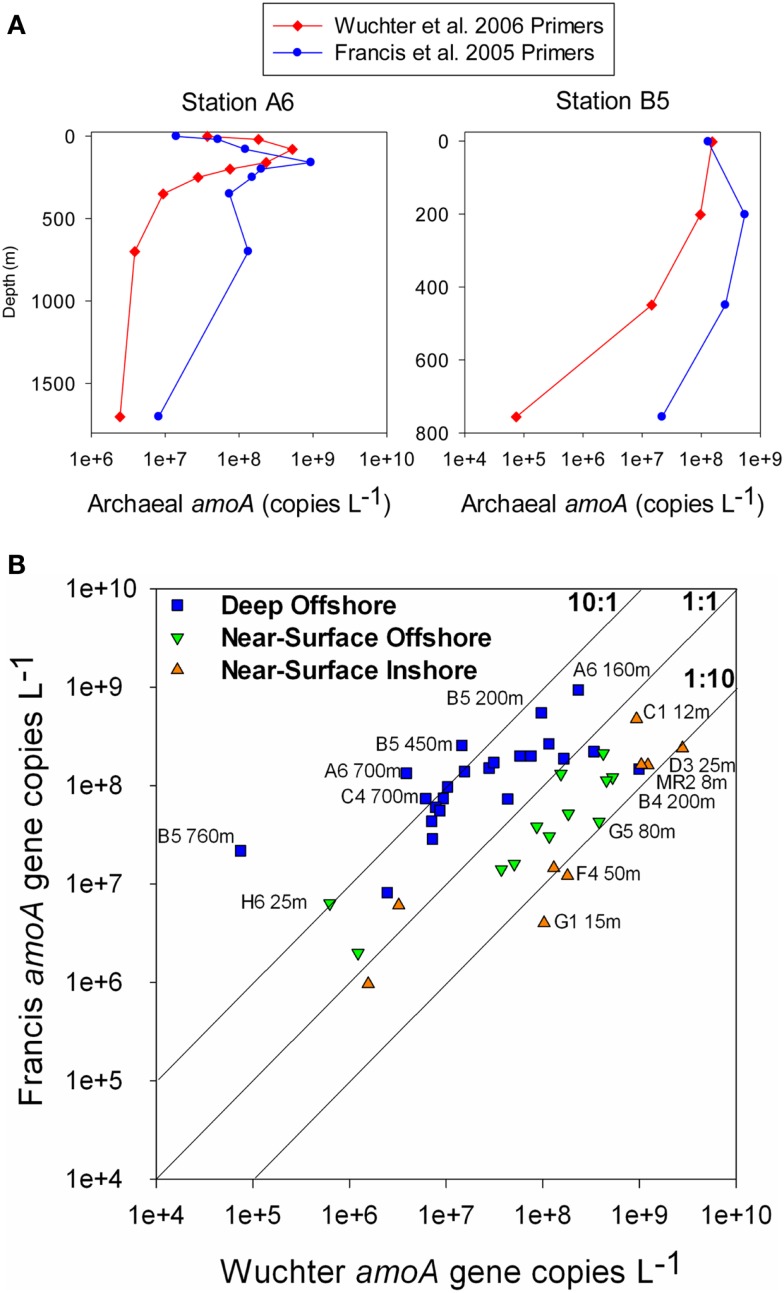
**Comparison of Archaeal *amoA* gene abundance estimated by qPCR reactions with primers from Wuchter et al. (2006) or Francis et al. (2005)**. **(A)** Profiles of *amoA* abundance at Stations A6 and B5 obtained using each primer set. 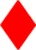 = Wuchter, 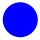 = Francis. **(B)** Abundance of *amoA* genes estimated using Francis primers versus abundance estimated using the Wuchter primers. “Deep,” >100 m sample depth; “near-surface,” = ≤ 100 m sample depth; “inshore,” = above continental shelf; “offshore,” = shelf break and beyond. **(A)** Station profiles of *amoA* quantified with different primer sets **(B)** *amoA* quantified by Wuchter et al. (2006) primers versus Francis et al. (2005) primers.

The abundance of Archaeal *amoA* genes reported in this study (up to 10^8^ copies L^−1^) is comparable to abundances reported for other continental shelf regions (Galand et al., 2006; Mincer et al., 2007; Kalanetra et al., 2009; Santoro et al., 2010), in the mesopelagic Pacific Ocean (Church et al., 2010), and in hypoxic zones (Beman et al., 2008; Molina et al., 2010). Differences in estimates of *amoA* abundance depended on the primer set used. Previous studies using the Wuchter primers reported low abundance of *amoA* relative to *rrs* in deep waters (Agogué et al., 2008; De Corte et al., 2009) compared to studies that used the Francis primers (Beman et al., 2010; Church et al., 2010; Santoro et al., 2010), suggesting that the Wuchter primers are biased against deep water clades of AOA. Our study supports these conclusions, but we also found that the Francis primers underestimated *amoA* abundance relative to *rrs* in surface water samples (Figure A6 in Appendix). Comparisons of primer sequences to alignments of *amoA* sequences from this study show single base-pair differences within Wuchter primer binding sites that could affect primer annealing and thus amplification (Figure A8 in Appendix). Our findings support the use of two different primer sets for the quantification of Archaeal *amoA* in near-surface versus deep water samples, as recommended by Beman et al. (2008). Alternatively, Thaumarchaeota abundance in DNA extracted from our samples estimated by qPCR of *rrs* agreed well with an independent assessment based on pyrosequencing. This suggests that the 334F/534R *rrs* primer set originally proposed by Suzuki et al. (2000) for quantifying Marine Group 1 Archaea may be more robust than *amoA* primer sets for quantifying Thaumarchaeota.

The *accA* gene, a proposed marker for archaeal autotrophy, was found at abundances almost equal to Thaumarchaeota *rrs* and *amoA* (amplified by the Francis primers) below 100 m depth (Table 2), in agreement with findings from the original *accA* survey of the Tyrrhenian Sea (Yakimov et al., 2009). *accA* was least abundant in surface water samples (2–70 m depth; e.g., Figure 2), especially at inshore stations and in the Mississippi River. A similar trend has been reported for South China Sea samples, where *accA* approached the limit of detection in samples <100 m (Hu et al., 2011). Since the *accA* primers were designed using a very small database, the apparent discrepancy between *accA* and Thaumarchaeota *rrs* abundance in near-surface samples may be due to the presence of populations in surface waters with divergent *accA* that are not detected by this primer set.

### Community composition

**Figure 9:**
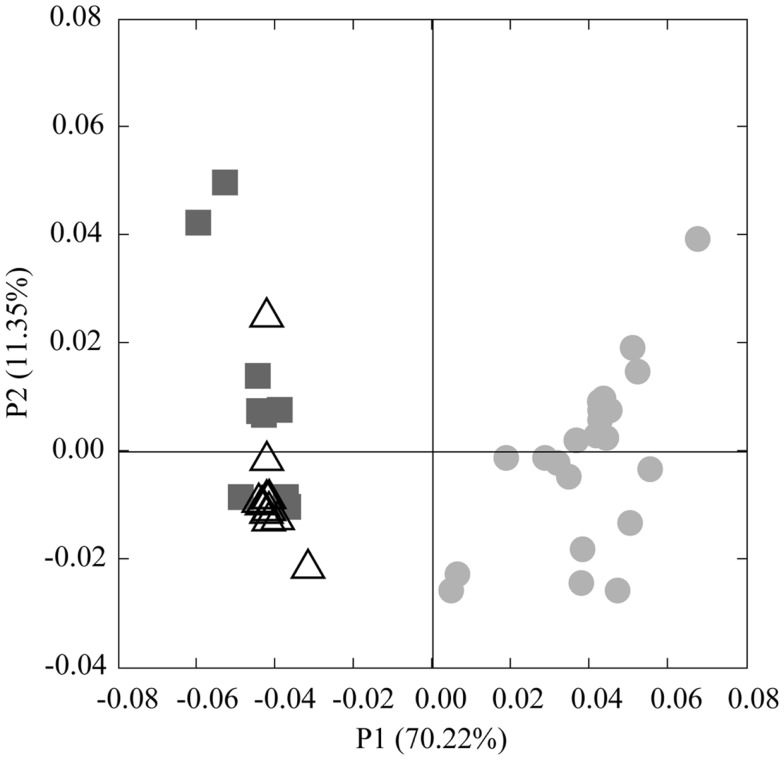
**Principal Coordinates Analysis (PCoA) of Thaumarchaeota *rrs* sequences obtained from 40 samples taken in the northern Gulf of Mexico (excluding Station MR1)**. Shapes indicate sample groupings: dark gray squares, deep, offshore; open triangles, near-surface, offshore; light gray circles, near-surface, inshore. The percentage of the variance explained by an axis is given in parentheses next to the axis title.

**Figure A7:**
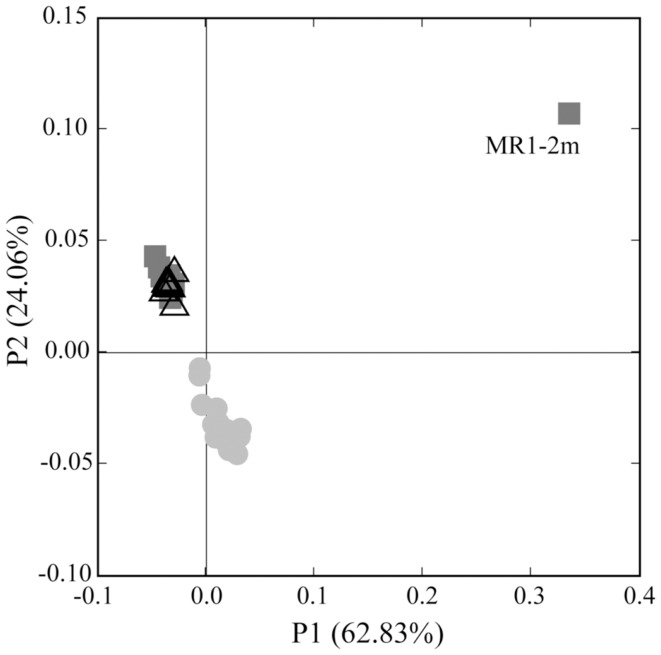
**Principal coordinates analysis (PCoA) of Thaumarchaeota *rrs* sequences obtained through 454 pyrosequencing of 41 samples and clone libraries generated from two depths at Station D5**. Shapes indicate sample groupings: dark gray squares = deep, offshore; open triangles = near-surface, offshore; light gray circles = near-surface, inshore. The percentage of the variance explained by an axis is given in parentheses next to the axis title.

**Figure A8:**
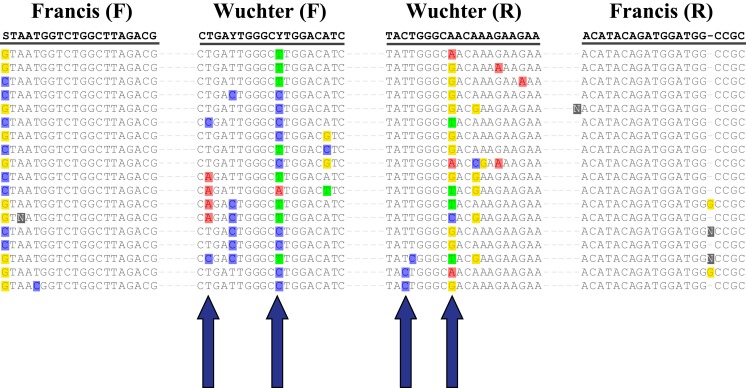
**Mismatches between *amoA* primer sequences and environmental sequences retrieved from samples taken in the northern Gulf of Mexico**. Sequences shown were collected from Station D5, 200 m depth, and were trimmed to regions complementary to the Wuchter et al. (2006) and Francis et al. (2005) primer sets. The top line represents the consensus sequence, while arrows indicate key differences between environmental and primer sequences.

We identified a number of clades that appear to be unique to the northern Gulf of Mexico. These were seen in *rrs* genes from both clone libraries and pyrosequencing reads (e.g., D5–200 m-66 [KC330801], -71 [KC330804], -85 [KC330810]; D5–100 m-15 [KC330788]; Figure 5), in *amoA* gene sequences (e.g., D5–100 m-amoA-21 [KC349156], -35 [KC349170], -41 [KC349176], -51 [KC349185]; D5–200 m-amoA-30 [KC349251], -44 [KC349264]; Figure A1A in Appendix), and in *accA* gene sequences (e.g., D5–2 m-accA-05 [KC349402], -44 [KC349436]; D5–50 m-accA-53 [KC349545]; D5–100 m-accA-21 [KC349333], -29 [KC349340], -47 [KC349355]; D5–200 m-accA-11 [KC349365], -27 [KC349380], -36 [KC349389], -41 [KC349393]; D5–450 m-accA-20 [KC349475], -26 [KC349480]; Figure A1B in Appendix). Since the global distribution of *accA* genes has not been thoroughly surveyed, it is difficult to determine whether these clades are indeed unique to the Gulf of Mexico. Generally, the sub-populations of Thaumarchaeota represented by distinct OTUs of each gene grouped according to sample depth, with the most stringent segregation by depth observed for *rrs* and *accA*, which segregated as deep (200 and 450 m) and near-surface (2, 50, and 100 m) OTUs, as has been observed elsewhere for *amoA* (Francis et al., 2005; Beman et al., 2008, 2010; Kalanetra et al., 2009; Church et al., 2010; Santoro et al., 2010). Archaeal *amoA* phylotypes retrieved from Station D5 were also distributed according to sample depth (Figure 6A), with a near-surface “Group A” and deep “Group B” (Francis et al., 2005). Since these distributions of each of these genes were determined by independent PCR amplifications, it is not possible to directly associate *rrs*, *amoA*, and *accA* genotypes in our samples; however, the coincident groupings of these three markers of completely different physiological functions suggest differentiation of these Thaumarchaeota populations at a genomic level. Unifrac analysis suggests that Thaumarchaeota populations at these stations resolve into three sub-populations, segregated by depth and by factors covarying with depth, with strongest separation between surface (depth < 100 m) and deep water populations (Figure 9; Figures A4 and A7 in Appendix).

A few of the *accA* gene sequences retrieved from Station D5 clustered with previously defined ecotypes of the “Deep Water *accA* Clade” (Yakimov et al., 2009, 2011), referred to here as Deep Ecotypes 1a, 1b, and 2 (Figure 6B). Inferred amino acid sequences of all but 8 of the 87 *accA* amplicons we retrieved from 200 and 450 m grouped into Deep Ecotype 2. No representatives of Deep Ecotypes 1a or 1b were identified, although a group of more divergent sequences similar to these ecotypes was evident (Figure 6B). Since previous studies concentrated on samples from deeper waters, we have added Near-Surface Ecotypes 1a and 1b to the “Shallow Water *accA* Clade” (Yakimov et al., 2011). Both of the Sargasso Sea reference sequences from this clade fit into Ecotype 1a, which contained only sequences from near-surface waters (≤100 m) of the northern Gulf of Mexico. The *accA* sequence from “*Ca*. *Nitrosopumilus maritimus*” SCM1 (Walker et al., 2010) grouped with marine sediment clones and with “*Ca. Nitrosoarchaeum limnia*” SFB1 (Blainey et al., 2011); we have thus allocated these sequences to a “*Nitrosopumilus*-like group.” We also note a distinct lineage of *accA* (OTU 2, “Near-Surface Ecotype 1b”; Figure 6B) containing sequences from the northern Gulf of Mexico and the South China Sea (“Shallow group II” in Hu et al., 2011).The sequences we retrieved extend coverage of the diversity of *accA* environmental sequences to near-surface sites and provide additional references for refining ecotype characterizations as more sequences are added to the databases.

## Conclusion

AOA and Thaumarchaeota were abundant in the northern Gulf of Mexico coastal waters we sampled, accounting for up to 40% (qPCR) or 54% (pyrosequencing) of the total bacterioplankton population and outnumbering AOB by 10- to 100-fold. The ratio of AOA to NOB in our samples was lower than reported in other studies, suggesting that other pathways for nitrite oxidation may be more important in the northern Gulf of Mexico than elsewhere. A diverse community of Thaumarchaeota was observed at Station D5 near the Mississippi River plume in clone libraries constructed from archaeal genes of interest (*rrs*, *amoA*, and *accA*), with clades that seem to be unique to waters of the northern Gulf of Mexico. Consistent with this observation, and in contrast to studies of many other coastal waters, the *amoA* sequence most similar to Nmar_1500, the *amoA* gene from “*Ca. N. maritimus*” strain SCM1, was only 91% similar. Through analysis of *rrs* sequences generated using 454 pyrosequencing, we observed distinct clades of Thaumarchaeota that were distributed primarily by depth, with clear differences between near-surface (≤100 m) and deep (>100 m) populations. The distribution of *rrs* sequences in clone libraries generated from samples collected at Station D5 was consistent with this pattern, suggesting that parallel differences in the composition of Thaumarchaeota populations defined by other genes observed at this station were applicable to the rest of the northern Gulf of Mexico. Finally we found correlations between abundances of Thaumarchaeota genes in this region and environmental variables depth, temperature, turbidity, pH, and oxygen; however, the manner in which these variables influence Thaumarchaeota metabolism and thus distribution remains unclear.

## Conflict of Interest Statement

The authors declare that the research was conducted in the absence of any commercial or financial relationships that could be construed as a potential conflict of interest.

## Footnotes

^1^http://oceancolor.gsfc.nasa.gov

^2^www.geneious.com/

^3^http://tree.bio.ed.ac.uk/

^4^metagenomics.anl.gov

^5^http://www.mothur.org/wiki/Silva_reference_files

^6^http://cran.r-project.org/web/packages/lmodel2/index.html

## Appendix

### Methods

#### qPCR standards

Standards for qPCR reactions were constructed as in Kalanetra et al. (2009). Briefly, environmental DNA was amplified using gene-specific sequencing primers (Thaumarchaeota *rrs*, Archaeal *amoA*, Bacterial *amoA*) or qPCR primers (*accA*) under standard PCR conditions. For Bacterial *rrs* qPCR, *E. coli* genomic DNA was used. The resulting PCR product was loaded onto an agarose gel, electrophoresed, and a band of expected product size was excised. This band was purified using the QIAquick^®^ Gel Extraction Kit (QIAGEN) and cloned into *E. coli* TOP10 chemically competent cells after insertion into a TOPO 4 vector (Invitrogen) using the manufacturer’s instructions. Clones were selected at random and sequenced to check insert specificity. Those with positive insertions were grown overnight in LB broth with ampicillin, and plasmids were extracted using the QIAprep Spin Miniprep Kit^®^ (QIAGEN). Plasmids were linearized using the restriction enzyme *Not*I (New England Biolabs), then purified in the same manner as PCR products above. Concentrations of linearized plasmid DNA were measured with the Quant-iT™ PicoGreen^®^ dsDNA reagent (Invitrogen) using a Picofluor handheld fluorometer (Turner Designs). Gene concentration calculations were based on measured DNA concentrations, plasmid length, and insert sequence length. Standards were then diluted to a range of 10^7^–10^1^ copies μL^−1^ for each reaction.

#### Thaumarchaeota *hcd* gene assay

In addition to *accA*, another gene in the 3-hydroxypropionate/4-hydroxybutyrate pathway, *hcd*, encoding the enzyme 4-hydroxybutyryl-CoA dehydratase, has been suggested as a potential marker for carbon fixation in Thaumarchaeota (Offre et al., 2011). Primers for this gene have been developed and tested on soil Thaumarchaeota populations (Offre et al., 2011). We explored using these primers to quantify *hcd* abundance in our samples. We were unable to obtain the desired amplification specificity with these primers and our samples (determined by agarose gel electrophoresis then cloning and sequencing putative amplicons, see below).

#### Gene abundance and ratio calculations

The number of gene copies detected by qPCR (copies per reaction) was converted to environmental concentrations (copies L^−1^) using the original sample volume filtered (∼1 L), the portion of the lysate purified (800 of 2000 μL), the final volume of the purified extract (50 μL, we also measured DNA concentration in this extract), and the portion of the purified DNA extract used in each qPCR reaction (2 μL). This calculation assumes that all bacterioplankton cells were collected on the filter, that the DNA contribution from eukaryotes was negligible, that all of the DNA from all of the cells collected on each filter was released into the lysate, then extracted and purified from the lysate and detected by qPCR with 100% efficiency by our methods (see discussion and calibration in Kalanetra et al., 2009). The contribution of Thaumarchaeota to the prokaryotic population was estimated from Thaumarchaeota and Bacteria *rrs* abundance by assuming 1.8 *rrs* per Bacteria genome (Biers et al., 2009), 1.0 *rrs* per Thaumarchaeota genome (IMG database) or 2.0 *rrs* per NOB genome (Mincer et al., 2007). Thaumarchaeota abundance was then divided by the total prokaryotic abundance (Bacteria plus Thaumarchaeota; Euryarchaeota were present in some samples but were never abundant, see below, and were not measured by qPCR) to calculate the contribution of Thaumarchaeota cells to the prokaryotic community. Ratios of gene abundance in a given sample were calculated directly from the qPCR data (copies μL^−1^ of extract).

#### BEST analysis

BEST analysis was performed for all samples collected in addition to the subset of samples for which nutrient data were available. Nutrient data were collected by researchers interested in modeling phytoplankton growth and thus were only available for near-surface samples. For these samples, gene abundances were log-transformed and resemblance distances for each gene between samples were calculated using Bray–Curtis similarity; resemblances for environmental data were calculated using the Euclidean distance. The resultant similarity matrices were combined and analyzed with Biota and/or Environment matching (BioEnv) through the BEST (Clarke, 1993) procedure in PRIMER (Clarke and Gorley, 2006). The significance of BEST results for each gene was tested using 999 permutations, and the null hypothesis of no species-environment relationship was rejected for all results with *p* ≤ 0.001.

### Results

#### Gene ratios

Ratios of archaeal *amoA*:Thaumarchaeota *rrs* ranged from 0.001 (B5–760 m) to 6.6 (G1–15 m) when using the Wuchter primers to quantify Thaumarchaeota *amoA* (Table 2; Figure A5B). Low ratios of *amoA*:*rrs* seemed to coincide with deep (>100 m) samples (Table 2; Figure A5A). In contrast, ratios of *amoA*:*rrs* ranged from 0.002 to 1.9 with an average of 0.5 when Thaumarchaeota *amoA* abundance was estimated using the Francis primers. The Francis primer set detected more *amoA* genes below 200 m depth, sometimes up to 1000 times more than the Wuchter primer set (Figure A6). In contrast, estimates of *amoA* abundance in near-surface (≤100 m) samples using the Wuchter primers were 10 to 100-fold greater than estimates based on the Francis primers (Figure A6). Ratios of *accA*:Thaumarchaeota *rrs* ranged from 0.0002 to 1.3 (Table 2). We detected the fewest copies of *accA* per Thaumarchaeota *rrs* in near-surface (≤100 m) samples (Figure A5B).

#### Thaumarchaeota *hcd* genes

*hcd* PCR products were also obtained using the primer set from Offre et al. (2011). However, the *hcd* primers yielded three bands of ∼200, ∼350, and ∼400 bp by agarose gel electrophoresis. Analysis of sequences from the ∼200 bp band indicated non-specific amplification, so these sequences are not considered further. Sequences from the ∼350 and ∼400 bp bands were most similar to *hcd* from Thaumarchaeota (BLASTx to the RefSeq database). Since non-specific amplification prevented reliable qPCR quantification of *hcd* in our samples, we did not pursue this marker further. The sequences obtained from ∼350 and ∼400 bp bands have been submitted to GenBank (NCBI) under accession numbers KC409223 to KC409237.

#### Community composition

As expected, phylogenetic analysis of *amoA* nucleotide sequences (Figure A1A) revealed more diversity than was apparent in inferred amino acid sequences, with 47 OTUs (97% similarity cutoff; Table A4) identified in the 100 and 200 m samples from Station D5. Seventeen of the 47 *amoA* OTUs only contained sequences from 100 m (1–22 sequences in each OTU), while 23 OTUs only contained sequences from 200 m (1–8 sequences in each). The *amoA* sequence most similar to either “*Candidatus Nitrosopumilus* *maritimus*” strain SCM1 or to *Nitrosopumilus* sp. NM25 was obtained from the 100 m library, and it was only 91% similar to either sequence.

**Table A1:** **Primers used in this study**.

Target gene*	Primer/probe**	Sequence (5′→3′)	Application	Detection limit	qPCR efficiency	Reference
Archaeal *rrs*	21F 958R	TTCCGGTTGATCCYGCCGGA YCCGGCGTTGAMTCCAATT	PCR and sequencing	N/A	N/A	DeLong (1992)
Thaumarchaeal *rrs*	G1_334F G1_554R	AGATGGGTACTGAGACACGGAC CTGTAGGCCCAATAATCATCCT	qPCR	4.08 × 10^3^ copies L^−1^	96.5−112.7%	Suzuki et al. (2000)
	TM519AR	TTACCGCGGCGGCTGGCAC				Suzuki et al. (2000)
pSL12 *rrs*	pSL12_750F pSL12_876R	GGTCCRCCAGAACGCGC GTACTCCCCAGGCGGCAA	qPCR	1.07 × 10^4^ copies L^−1^	96.9−103.1%	Mincer et al. (2007)
Bacterial *rrs*	BACT1369F PROK1492R	CGGTGAATACGTTCYCGG GGWTACCTTGTTACGACTT	qPCR	1.14 × 10^4^ copies L^−1^	91.6−113.2%	Suzuki et al. (2000)
	TM1389F	CTTGTACACACCGCCCGTC	
Universal *rrs*	515F	GCCTTGCCAGCCCGCTCAG GTGTGCCAGCMGCCGCGGTAA	pyrosequencing	N/A	N/A	King et al. (2013)
	806R^#^	GCCTCCCTCGCGCCATCAGNN NNNNNNNNNNGGGGACTACV SGGGTATCTAAT	
Archaeal *amoA* (W^+^)	Arch-amoA-for Arch-amoA-rev	CTGAYTGGGCYTGGACATC TTCTTCTTTGTTGCCCAGTA	qPCR	1.44 × 10^4^ copies L^−1^	95.1−103.6%	Wuchter et al. (2006)
Archaeal *amoA* (F^+^)	ArchamoAF ArchamoAR	STAATGGTCTGGCTTAGACG GCGGCCATCCATCTGTATGT	qPCR and sequencing	1.79 × 10^4^ copies L^−1^	88.4−96.0%	Francis et al. (2005)
Bacterial *amoA*	amoA-1F amoA-r New	GGGGTTTCTACTGGTGGT CCCCTCBGSAAAVCCTTCTTC	qPCR	1.63 × 10^4^ copies L^−1^	88.4−95.6%	Rotthauwe et al. (1997)
						Hornek et al. (2006)
Archaeal *accA*	Crena_529F Crena_981R	GCWATGACWGAYTTTGTYRTAATG TGGWTKRYTTGCAAYTATWCC	qPCR and sequencing	1.25 × 10^4^ copies L^−1^	80.5−91.1%	Yakimov et al. (2009)
Archaeal *hcd*	hcd-465F (S) hcd-911F (Q)	GGHGGTGCWATGACTGAT AGCTATGTBTGCAARACAGG	PCR, qPCR, sequencing	N/A	N/A	Offre et al. (2011)
	hcd-1267R (S,Q)	CTCATTCTGTTTTCHACATC	

**rrs, 16S rRNA gene; amoA, ammonia monooxygenase gene, Bacteria amoA primers only amplify amoA genes from β-Proteobacteria; accA, biotin-dependent acetyl-CoA/propionyl-CoA carboxylase gene; hcd, 4-hydroxybutyryl-CoA dehydratase*.

***(S), Sequencing, (Q), qPCR, TM, TaqMan Probe*.

^+^*Archaeal amoA (W) and (F) refer to Wuchter et al. (2006) and Francis et al. (2005) primer sets, respectively (as mentioned in text)*.

*^#^For primer 806R, N’s in sequence = barcode sequence region*.

**Table A2:** **qPCR estimates of the abundance of *rrs*, *amoA*, and *accA* genes in samples from the northern Gulf of Mexico**.

Station ID	Depth (m)	Amount filtered (L)	Wuchter Arch *amoA* copies/L	Francis Arch *amoA* copies/L	Arch *accA* copies/L	Thaum *rrs* copies/L	pSL12 *rrs* copies/L	AOB *amoA* copies/L	Bacteria *rrs* copies/L	Thaum% of total prokarya	
A2	18	1.0	ND	ND	ND	5.85E + 05	*1.12E* + *02*	ND	4.60E + 08	0.23	
A4	17	1.2	ND	ND	ND	2.08E + 06	ND	ND	8.70E + 08	0.43	
A4	43	1.2	ND	ND	ND	2.20E + 06	ND	ND	4.32E + 08	0.91	
A6	2	1.1	2.32E + 06	8.82E + 05	*6.48E* + *02*	2.62E + 06	*4.50E* + *01*	*2.79E* + *02*	5.27E + 08	0.89	
A6	20	1.1	1.15E + 07	3.24E + 06	1.33E + 05	9.52E + 06	*1.72E* + *03*	*5.23E* + *03*	1.02E + 09	1.66	
A6	80	1.1	3.29E + 07	7.61E + 06	3.30E + 05	1.81E + 07	*1.52E* + *03*	*7.07E* + *03*	3.48E + 08	8.54	
A6	160	1.1	1.46E + 07	5.86E + 07	1.80E + 07	5.45E + 07	1.32E + 04	*8.80E* + *03*	2.42E + 08	28.88	
A6	200	1.1	4.72E + 06	1.25E + 07	5.83E + 06	2.00E + 07	*6.57E* + *03*	*1.43E* + *03*	1.77E + 08	16.94	
A6	250	1.1	1.74E + 06	9.41E + 06	8.56E + 06	2.34E + 07	*5.98E* + *03*	*1.03E* + *03*	1.28E + 08	24.80	
A6	350	1.1	5.86E + 05	4.64E + 06	3.95E + 06	8.86E + 06	*4.50E* + *03*	*5.50E* + *02*	4.46E + 07	26.36	
A6	700	1.1	2.42E + 05	8.36E + 06	3.47E + 06	9.28E + 06	*3.61E* + *03*	LD	2.49E + 07	40.17	
A6	1700	1.2	1.53E + 05	5.11E + 05	1.74E + 06	3.23E + 06	*1.05E* + *04*	ND	4.31E + 07	11.92	
B4	200	1.1	6.20E + 06	9.20E + 06	1.56E + 07	2.28E + 07	1.30E + 04	*3.52E* + *03*	3.30E + 08	11.06	
B4	530	1.2	6.45E + 05	6.05E + 06	5.46E + 06	1.42E + 07	*5.78E* + *03*	*5.24E* + *03*	1.08E + 08	19.11	
B5	2	1.1	9.62E + 06	8.21E + 06	5.54E + 05	8.50E + 06	*2.71E* + *03*	*4.71E* + *03*	6.07E + 08	2.46	
B5	200	1.1	6.05E + 06	3.43E + 07	1.21E + 07	3.25E + 07	3.52E + 04	*1.92E* + *03*	3.68E + 08	13.72	
B5	450	1.1	9.08E + 05	1.60E + 07	1.32E + 07	3.38E + 07	1.54E + 04	*2.27E* + *03*	2.04E + 08	23.01	
B5	760	1.1	*4.65E* + *03*	1.36E + 06	ND	3.48E + 06	*4.17E* + *03*	*1.34E* + *02*	4.40E + 07	12.49	
C1	12	1.1	1.74E + 08	1.50E + 07	9.53E + 05	1.10E + 08	LD	2.10E + 06	1.08E + 10	1.79	
C4	2	1.0	ND	ND	ND	1.76E + 06	LD	ND	6.03E + 08	0.52	
C4	200	1.0	7.21E + 06	1.66E + 07	1.00E + 07	4.93E + 07	2.39E + 04	*3.35E* + *03*	2.04E + 08	30.29	
C4	700	1.0	3.84E + 05	4.62E + 06	7.17E + 06	1.26E + 07	1.21E + 04	*2.35E* + *03*	4.33E + 07	34.30	
D3	25	1.1	7.66E + 07	1.01E + 07	5.10E + 05	1.47E + 07	*7.17E* + *03*	2.54E + 05	6.60E + 08	3.85	
D3	68	1.1	8.13E + 06	7.53E + 05	*1.02E* + *03*	ND	*5.27E* + *02*	*1.53E* + *04*	3.11E + 05	0.30	
D5	2	1.1	7.31E + 06	1.91E + 06	1.46E + 05	4.71E + 06	*6.21E* + *02*	*4.55E* + *02*	7.34E + 08	1.14	
D5	50	1.1	5.42E + 06	2.40E + 06	3.14E + 05	5.37E + 06	*1.64E* + *03*	*1.29E* + *03*	8.93E + 08	1.07	
D5	100	1.1	2.85E + 07	7.08E + 06	1.36E + 06	2.14E + 07	*3.61E* + *03*	*2.28E* + *03*	3.89E + 08	9.00	
D5	200	1.2	1.94E + 06	1.08E + 07	1.08E + 07	1.69E + 07	*8.86E* + *03*	*2.77E* + *03*	1.23E + 08	19.84	
D5	350	1.1	9.70E + 05	8.69E + 06	1.39E + 07	2.45E + 07	*6.54E* + *03*	*1.49E* + *03*	8.53E + 07	34.08	
D5	450	1.0	4.88E + 05	3.76E + 06	7.18E + 06	1.08E + 07	*4.47E* + *03*	*5.24E* + *03*	3.56E + 07	35.34	
D5	900	1.1	4.43E + 05	2.72E + 06	6.84E + 06	7.50E + 06	*6.27E* + *03*	*2.34E* + *03*	5.50E + 07	19.72	
E2	6	1.0	ND	LD	ND	ND	ND	ND	4.50E + 09	0.00	
E6	200	1.1	3.63E + 06	1.25E + 07	1.55E + 07	1.57E + 07	1.63E + 04	*4.83E* + *03*	1.80E + 08	13.56	
E6	800	1.1	5.40E + 05	3.49E + 06	6.05E + 06	4.73E + 06	*5.30E* + *03*	*1.14E* + *03*	1.49E + 08	5.41	
F4	50	1.1	1.12E + 07	7.60E + 05	7.42E + 04	5.92E + 06	*9.58E* + *02*	4.23E + 04	5.17E + 08	2.02	
F6	2	1.1	ND	ND	ND	4.79E + 04	LD	ND	4.08E + 08	0.02	
F6	200	1.2	1.03E + 07	1.17E + 07	1.75E + 07	1.56E + 07	1.23E + 04	*3.18E* + *03*	2.59E + 08	9.80	
F6	950	1.1	4.52E + 05	1.79E + 06	5.42E + 06	5.19E + 06	*5.43E* + *03*	*2.78E* + *03*	3.52E + 07	20.99	
G1	15	1.1	6.46E + 06	2.50E + 05	*9.09E* + *02*	*9.80E* + *05*	*6.41E* + *02*	2.37E + 04	9.27E + 08	0.19	
G5	80	1.1	2.39E + 07	2.69E + 06	5.93E + 05	9.77E + 06	*2.56E* + *03*	*6.09E* + *03*	6.20E + 08	2.76	
H1	7	1.0	3.79E + 05	*9.28E* + *02*	7.73E + 04	5.17E + 05	5.93E + 04	5.14E + 04	5.68E + 09	0.02	
H3	20	1.2	9.74E + 04	6.01E + 04	ND	1.37E + 05	*1.75E* + *02*	ND	6.74E + 08	0.04	
H6	2	1.1	7.64E + 04	1.24E + 05	*3.16E* + *02*	4.45E + 05	*4.14E* + *01*	ND	1.04E + 09	0.08	
H6	25	1.1	3.91E + 04	3.95E + 05	ND	2.05E + 05	*3.41E* + *02*	*1.67E* + *02*	1.34E + 09	0.03	
H6	45	1.1	3.18E + 06	1.00E + 06	2.67E + 04	1.86E + 06	*6.21E* + *02*	*1.02E* + *03*	9.42E + 08	0.35	
H6	80	1.1	2.64E + 07	1.33E + 07	2.79E + 06	1.59E + 07	*3.98E* + *03*	*5.64E* + *03*	4.72E + 08	5.74	
H6	110	1.1	2.12E + 07	1.39E + 07	1.48E + 06	2.03E + 07	*8.10E* + *03*	*8.18E* + *03*	3.17E + 08	10.33	
H6	200	1.2	2.16E + 06	ND	ND	1.66E + 07	ND	*1.23E* + *03*	1.13E + 08	20.98	
H6	280	1.1	2.71E + 06	4.57E + 06	2.16E + 06	4.80E + 06	*2.92E* + *03*	*2.29E* + *03*	1.83E + 09	0.47	
MR1	2	0.6	2.02E + 05	3.79E + 05	2.02E + 05	ND	3.30E + 05	7.44E + 05	1.26E + 10	0.00	
MR2	8	1.1	6.52E + 07	1.02E + 07	4.24E + 05	2.96E + 07	7.68E + 04	2.83E + 05	7.43E + 09	0.71	
MR3	110	1.0	5.82E + 07	2.97E + 07	1.00E + 07	4.60E + 07	2.93E + 04	1.50E + 05	1.78E + 09	4.46	

*ND, Not determined; qPCR abundance undetectable for this specific gene in this sample*.

*LD, Limit of detection; sample ran below limit of detection with high variability in assay*.

**Note that some values shown below the limit of detection (italicized) by our assay are included here because these values had low standard deviation in replicates*.

**Table A3:** **Abundance of Thaumarchaeota, Euryarchaeota, Nitrite-Oxidizing Bacteria (NOB), and Ammonia-Oxidizing Bacteria (AOB) *rrs* sequences in pyrosequenced libraries from DNA samples collected in the northern Gulf of Mexico**.

Station ID	Depth	Thaumarchaeota as a % of prokaryotes by qPCR of *rrs* (%)	Total number of *rrs* sequences in pyrosequenced library	Bacteria *rrs* hits in pyrosequenced libraries	Thaumarchaeota *rrs* hits in pyrosequenced libraries	Euryarchaeota *rrs* hits in pyrosequenced libraries	Nitrite-oxidizing bacteria *rrs* hits in pyrosequenced libraries	Ammonia-oxidizing bacteria *rrs* hits in pyrosequenced libraries	Thaumarchaeota as a % of prokaryotes by pyrosequencing *rrs* amplicons	Euryarchaeota as a % of prokaryotes by pyrosequencing *rrs* amplicons	Nitrite-oxidizing bacteria as a % of prokaryotes by pyrosequencing *rrs* amplicons	Ammonia-oxidizing bacteria as a % of prokaryotes by pyrosequencing *rrs* amplicons
A2	18	0.23	2297	2283	11	3	0	0	0.86	0.23	0.00	0.00
A4	17	0.43	9146	8947	71	128	3	1	1.41	2.54	0.03	0.02
A4	43	0.91	6185	5131	727	327	63	0	20.32	9.14	0.88	0.00
A6	2	0.89	6288	5888	111	289	2	0	3.28	8.54	0.03	0.00
A6	20	1.66	8487	7626	164	697	5	0	3.73	15.84	0.06	0.00
A6	80	8.53	8021	7132	583	306	36	1	12.83	6.73	0.40	0.02
A6	160	28.86	5787	3578	1779	430	121	0	47.23	11.42	1.61	0.00
A6	350	26.38	3552	2415	992	145	24	0	42.51	6.21	0.51	0.00
A6	700	40.18	3428	1993	1287	148	20	0	53.75	6.18	0.42	0.00
A6	1700	11.91	879	626	232	21	2	0	40.02	3.62	0.17	0.00
B4	200	11.05	8564	6925	1383	256	88	0	26.44	4.89	0.84	0.00
B4	530	19.06	5539	4243	1154	142	17	0	32.87	4.04	0.24	0.00
B5	2	2.46	764	683	12	69	0	0	3.07	17.63	0.00	0.00
B5	200	13.72	4053	3191	698	164	51	0	28.25	6.64	1.03	0.00
B5	450	23.07	4787	3304	1301	182	30	0	41.48	5.80	0.48	0.00
B5	760	12.51	5041	3891	1061	89	15	0	32.92	2.76	0.23	0.00
C4	2	0.52	7792	7654	127	11	0	0	2.90	0.25	0.00	0.00
C4	200	30.33	5898	4277	1359	262	62	1	36.38	7.01	0.83	0.03
C4	700	34.40	3587	2388	1083	116	13	0	44.94	4.81	0.27	0.00
D3	25	3.84	7369	6157	751	461	53	1	18.00	11.05	0.64	0.02
D3	68	0.30	7208	6231	720	257	2	0	17.22	6.15	0.02	0.00
D5	2	1.14	6892	6659	137	96	2	0	3.57	2.50	0.03	0.00
D5	50	1.07	7442	7033	134	275	1	1	3.32	6.80	0.01	0.02
D5	100	9.01	6019	5161	538	320	24	0	15.80	9.40	0.35	0.00
D5	350	34.12	2960	1835	979	146	23	0	48.99	7.31	0.58	0.00
D5	450	35.39	1572	945	573	54	10	0	52.19	4.92	0.46	0.00
D5	900	19.72	3574	2596	857	121	9	0	37.27	5.26	0.20	0.00
E2	6	0.00	8486	7953	444	89	16	0	9.13	1.83	0.16	0.00
E6	200	13.60	4454	3334	924	196	67	1	33.28	7.06	1.21	0.04
E6	800	5.41	6293	5659	570	64	5	0	15.35	1.72	0.07	0.00
F6	2	0.02	6839	6651	56	132	2	0	1.49	3.52	0.03	0.00
F6	200	9.79	6863	4953	1445	465	115	0	34.43	11.08	1.37	0.00
F6	950	21.04	4945	3800	1036	109	12	0	32.92	3.46	0.19	0.00
H1	7	0.02	4997	4942	2	53	0	0	0.07	1.93	0.00	0.00
H3	20	0.04	1179	1131	8	40	0	0	1.26	6.29	0.00	0.00
H6	2	0.08	7112	7030	6	76	0	0	0.15	1.94	0.00	0.00
H6	110	10.30	5364	4195	872	297	115	0	27.23	9.27	1.80	0.00
H6	280	0.47	7367	6022	1051	294	75	0	23.91	6.69	0.85	0.00
MR1	2	0.00	5265	5259	4	2	4	7	0.14	0.07	0.07	0.24
MR2	8	0.71	5759	5607	90	62	4	1	2.81	1.93	0.06	0.03
MR3	110	4.46	3356	2849	417	90	36	0	20.85	4.50	0.90	0.00
Total			221410	188177	25749	7484	1127	14				
Average			5400	4590	628	183	27	0	21.33	5.83	0.41	0.01
Max			9146	8947	1779	697	121	7	53.75	17.63	1.80	0.24
Min			764	626	2	2	0	0	0.07	0.07	0.00	0.00
Count	41	41	41	41	41	41	41	41				

**Table A4:** **Diversity indices for sequenced clones obtained from Station D5 calculated using mothur (v. 1.21.1; Schloss et al., 2009)**.

	Observed	Chao	ACE	Shannon	Simpson
*accA*	51	74.0	109	3.02	0.0862
*amoA*	47	86.4	107	3.19	0.0615
*amoA* 100m	22	35.2	55.7	2.39	0.139
*amoA* 200m	30	39.4	41.8	2.89	0.0801
*rrs*	10	11.0	12.8	1.30	0.451
*rrs* 100m	2	2.00	0.000	0.103	0.957
*rrs* 200m	9	10.0	11.8	1.96	0.141
*rrs* 454	2768	18700	57100	4.15	0.0654

*OTU similarity cutoffs were 2% (*rrs*) or 3% (*amoA*, *accA*). Statistics for *rrs* sequences (“*rrs* 454”) obtained from pyrosequencing are included for comparison*.

The *accA* nucleotide alignment contained 51 OTUs (97% similarity cutoff; Table A4) that clustered primarily by depth (Figure A1B). Sequences from deep samples (200 and 450 m) were assigned to 26 OTUs (1–13 sequences in each); only 4 of these OTUs contained any near-surface (2, 50, or 100 m) sequences. Twenty-one of the OTUs contained sequences exclusively from near-surface (≤100 m) samples (1–34 sequences in each). Almost half (101) of the sequences we retrieved were at least 77% similar to *accA* from “*Ca. N. maritimus*” strain SCM1; all of these were retrieved from near-surface waters except for six sequences from 200 m.

#### BEST analysis

Gene abundances determined by qPCR were compared to environmental data using the BEST procedure (Clarke, 1993). Results of this analysis (Table A7) show that abundances of Bacterial *amoA*, Archaeal *accA*, and pSL12 *rrs* – but not Bacterial *rrs* – were significantly correlated with fluorescence (chlorophyll a). Abundances of both Thaumarchaeota and Bacteria *rrs* were correlated with beam attenuation (turbidity), in combination with salinity and either fluorescence or temperature. Archaeal *amoA* abundance correlated with latitude, fluorescence, and salinity. Interestingly, BEST analysis (Table A7) showed that *amoA* abundance estimates obtained using the Wuchter et al. (2006) primers correlated with temperature (ρ = 0.442; *p* < 0.001), while *amoA* abundance estimated with the Francis et al. (2005) primers correlated with oxygen concentration (ρ = 0.474; *p* < 0.001).

**Table A5:** **Variables contributing to principal components axes**.

Variable	PC1	PC2
Latitude (°N)	−0.26	+0.30
Longitude (°W)	+0.066	+0.24
Depth (m)	+0.39	+0.21
Temperature (°C)	−0.36	−0.38
Salinity (PSU)	+0.19	−0.49
Dissolved oxygen (mg/L)	−0.36	−0.12
Rel. fluorescence (μg/L)	−0.39	+0.15
Beam attenuation (1/m)	−0.21	+0.44
pH (NBS)	−0.42	−0.32
Euphotic depth (m)	+0.31	−0.30

*Coefficients (values) are a measure of contribution of each variable to each of the principal component axes (PC1 and PC2) such that the higher the value, the greater the influence of the variable. A positive or negative sign represents the type of correlation each variable has on each axis. The total amount of variance explained by PC1 was 38.9% and 24.3% for PC2. Depth, water column depth; Rel. Fluorescence, Relative fluorescence, chlorophyll a equivalents; beam attenuation, turbidity; euphotic depth, photic zone depth*.

**Table A6:** **Results of CCA analysis of relationship between qPCR-estimated gene abundances and environmental data in the northern Gulf of Mexico**.

Axes	CCA1	CCA2	CCA3	CCA4
Eigenvalues	0.148	0.135	0.012	0.008
Gene-environment correlations	0.655	0.765	0.352	0.230
**CUMULATIVE PERCENTAGE VARIANCE**				
Of gene abundance data	17.0	32.6	34.0	34.9
Of gene-environment relation	47.9	91.7	95.7	98.3

*Values for all four canonical axes are shown, but only CCA1 and CCA2 were used to construct a biplot of the data (Figure 8)*.

**Table A7:** **Results of BEST analysis comparing gene abundance to **(A)** environmental factors and **(B)** environmental factors and nutrients (only near-surface samples used with nutrients)**.

Gene	Number of variables	Correlation (ρ*)	Contributing environmental variables	
**(A) BEST ANALYSIS WITH ENVIRONMENTAL VARIABLES**				
Thaumarchaeota *rrs*	3	0.507	Salinity, RF, beam attenuation	
pSL12 *rrs*	1	0.457	RF	
Bacterial *rrs*	3	0.613	Temperature, salinity, beam attenuation	
Archaeal *amoA* W	4	0.442	Latitude, temperature, salinity, RF	
Archaeal *amoA* F	4	0.474	Latitude, salinity, oxygen, RF	
Bacterial *amoA*	1	0.462	RF	
Archaeal *accA*	1	0.460	RF	

**Gene**	**Number of variables**	**Correlation (ρ*)**	***p*-Value^+^**	**Contributing environmental variables and nutrients**

**(B) BEST ANALYSIS WITH ENVIRONMENTAL VARIABLES AND NUTRIENTS**				
Thaumarchaeota *rrs*^#^	1	0.429	0.018	Beam attenuation
pSL12 *rrs*	5	0.337	0.102	Latitude, salinity, RF, nitrate, silicate
Bacterial *rrs*^#^	2	0.587	0.001	Beam attenuation, silicate
Archaeal *amoA* W	3	0.374	0.067	Latitude, RF, silicate
Archaeal *amoA* F^#^	2	0.374	0.010	Latitude, RF
Bacterial *amoA*	2	0.264	0.269	Latitude, RF
Archaeal *accA*	2	0.269	0.246	Latitude, RF

*BEST analysis (Clarke, 1993) performed with PRIMER v6 software (Clarke and Gorley, 2006). Archaeal *amoA* W, amplified with Wuchter et al. (2006) *amoA* primer set; Archaeal *amoA* F, amplified with Francis et al. (2005) *amoA* primer set; RF, relative fluorescence (chlorophyll a equivalents); beam attenuation, turbidity*.

**ρ in section A is the Spearman rank correlation coefficient where ρ > 0 rejects the null hypothesis; all results had a significance *p* ≤ 0.001, determined from 999 permutations*.

**ρ in section B is the Spearman rank correlation coefficient where ρ > 0 rejects the null hypothesis; p-values are given*.

*^+^*p* is the significance of the result, determined from 999 permutations*.

*^#^*p* ≤ 0.05*.

Nutrient data (including nitrite, nitrate, ammonia, phosphate, and silicate; provided by S. Lohrenz) were only available for near-surface samples. Gene abundances for Bacterial *rrs*, pSL12 *rrs*, and Archaeal *amoA* amplified with Wuchter primers (Table A7) correlated with silicate in combination with other variables, although only the Bacterial *rrs* result was significant (*p* < 0.001). Only the results with the highest Spearman’s rank correlation coefficient (ρ) are shown in Table A7; however, weaker correlations to nutrients were found with the second highest result. Archaeal *amoA* amplified with Francis primers (ρ = 0.446; *p* ≤ 0.010; data not shown) and Bacterial *rrs* (ρ = 0.583; *p* < 0.001; data not shown) were correlated with nitrate, while Thaumarchaeota *rrs* (ρ = 0.409; *p* ≤ 0.018; data not shown) also correlated with nitrite and silicate together.

### Discussion

#### Community composition

Almost all of the near-surface (≤100 m) Thaumarchaeota *rrs* sequences were ≥98% similar to the *rrs* from “*Ca. N. maritimus*” strain SCM1, as well as to *Nitrosopumilus* sp. NM25, retrieved from sand taken from a *Zostera* seagrass bed (Matsutani et al., 2011). The group containing these sequences included a sequence retrieved from cloned PCR amplicons sequenced from a tidal creek (the Duplin River) adjacent to Sapelo Island, Georgia (Hollibaugh et al., 2011), as well as “*Ca. Nitrosoarchaeum limnia*” strain SFB1 (Blainey et al., 2011), which was enriched from a sample taken in the oligohaline reach of North San Francisco Bay. This contrasts with clones recovered from 200 m in the northern Gulf of Mexico, where sequences were distributed among 9 OTUs, indicating a richer community of Thaumarchaeota (agreeing with the Shannon index of these samples calculated from pyrosequencing data; Table A4). We did not recover any clones related to the pSL12-like clade at Station D5, which is consistent with their low *rrs* abundance as estimated by qPCR.

A nucleotide alignment of *accA* genes from this study produced a phylogenetic tree (Figure A1B) that supported the groupings found in trees generated from inferred amino acid alignments (Figure 6B); however, some samples from Station D5 (mostly from 200 m depth) clustered with representatives from Deep Ecotype 1a (Yakimov et al., 2011) at the nucleotide level. Additionally, a novel deep cluster of sequences from the Gulf of Mexico and the South China Sea was identified (“Deep Ecotype 3”; Figure A1B).

#### Gene ratios

High ratios of *amoA*:Thaumarchaeota *rrs* genes at certain stations (Figure A5A; Table 2) could indicate a population of AOA with multiple *amoA* copies per genome or the presence of a group of Archaea that are not detected by the *rrs* primer set we used (e.g., Beman et al., 2008; Teske and Sorensen, 2008), but that contain a homolog of the *amoA* gene (for example the pSL12-like clade). The latter seems less likely for pSL12 in particular, given the low abundance of *rrs* from this group at most stations in the northern Gulf of Mexico. However, in the Mississippi River at station MR1 (salinity of 0), the abundance of pSL12 *rrs* genes was equal to Archaeal *amoA* gene abundance, regardless of the *amoA* primer set used, while Thaumarchaeota *rrs* genes were undetectable. Low ratios of *amoA*:*rrs* have been proposed to indicate a potential for heterotrophy in Thaumarchaeota (Agogué et al., 2008; De Corte et al., 2009; Kalanetra et al., 2009); however, this has yet to be confirmed definitively and may simply reflect depth-dependent shifts in sub-populations that affect our ability to quantify them by qPCR, as shown by Beman et al. (2008) and others.

Our data indicate that the ratio of *amoA*:*rrs* gene abundance decreases with depth; however, we also observed increases in the *accA*:*rrs* ratio for deeper waters (Figure A5B). The *amoA*:*accA*:*rrs* ratios we found are not consistent with the expected 1:1:1 ratio found in the “*Ca. N. maritimus*” strain SCM1 genome (Walker et al., 2010). In samples ≤100 m, this ratio is 1.8:0.1:1 or 0.5:0.1:1, while deeper samples show 0.2:0.6:1 or 0.6:0.6:1 depending on whether the Wuchter or Francis *amoA* primer sets were used (Table 2). In deeper waters where Thaumarchaeota *rrs* are most abundant, using the Francis primers produces ratios most similar to those found in “*Ca. N. maritimus*” strain SCM1. Direct comparison of *amoA* abundances in our samples as determined by the Wuchter versus Francis primer sets (Figure A6) demonstrate this clearly.

## References

[B84] Biers E. J. Sun S. L. Howard E. C. (2009). Prokaryotic genomes and diversity in surface ocean waters: interrogating the global ocean sampling metagenome. Appl. Environ. Microbiol. 75, 2221–2229 10.1128/AEM.02118-08 19201952 PMC2663191

[B85] Clarke K. R. (1993). Non-parametric multivariate analyses of changes in community structure. Aust. J. Ecol. 18, 117–143 10.1111/j.1442-9993.1993.tb00438.x

[B86] Hornek R. Pommerening-Roser A. Koops H. P. Farnleitner A. H. Kreuzinger N. Kirschner A. (2006). Primers containing universal bases reduce multiple amoA gene specific DGGE band patterns when analysing the diversity of beta-ammonia oxidizers in the environment. J. Microbiol. Methods 66, 147–155 10.1016/j.mimet.2005.11.001 16343671

[B87] Hollibaugh J. T. Gifford S. Sharma S. Bano N. Moran M. A. (2011). Metatranscriptomic analysis of ammonia-oxidizing organisms in an estuarine bacterioplankton assemblage. ISME J. 5, 866–878 10.1038/ismej.2010.172 21085199 PMC3105763

[B88] Rotthauwe J. H. Witzel K. P. Liesack W. (1997). The ammonia monooxygenase structural gene amoA as a functional marker: molecular fine-scale analysis of natural ammonia-oxidizing populations. Appl. Environ. Microbiol. 63, 4704–4712 9406389 10.1128/aem.63.12.4704-4712.1997 PMC168793

[B89] Teske A. Sorensen K. B. (2008). Uncultured archaea in deep marine subsurface sediments: have we caught them all? ISME J. 2, 3–18 10.1038/ismej.2007.90 18180743

